# In Silico Screening Accelerates Nanocarrier Design for Efficient mRNA Delivery

**DOI:** 10.1002/advs.202401935

**Published:** 2024-06-05

**Authors:** Tristan Henser‐Brownhill, Liam Martin, Parisa Samangouei, Aaqib Ladak, Marina Apostolidou, Benita Nagel, Albert Kwok

**Affiliations:** ^1^ Nuntius Therapeutics Limited London W10 5JJ UK

**Keywords:** drug delivery, LNP, machine learning, mRNA delivery, nanocarriers, nanoparticles, peptide dendrimers

## Abstract

Lipidic nanocarriers are a broad class of lipid‐based vectors with proven potential for packaging and delivering emerging nucleic acid therapeutics. An important early step in the clinical development cycle is large‐scale screening of diverse formulation libraries to assess particle quality and payload delivery efficiency. Due to the size of the screening space, this process can be both costly and time‐consuming. To address this, computational models capable of predicting clinically relevant physio‐chemical properties of dendrimer‐lipid nanocarriers, along with their mRNA payload delivery efficiency in human cells are developed. The models are then deployed on a large theoretical nanocarrier pool consisting of over 4.5 million formulations. Top predictions are synthesised for validation using cell‐based assays, leading to the discovery of a high quality, high performing, candidate. The methods reported here enable rapid, high‐throughput, in silico pre‐screening for high‐quality candidates, and have great potential to reduce the cost and time required to bring mRNA therapies to the clinic.

## Graphical abstract

A machine learning platform capable of predicting physio‐chemical properties and mRNA delivery efficiency of nanocarriers is developed to accelerate screening of millions of formulations in silico. Synthesis and evaluation of predictions led to the discovery of a high‐quality candidate, supporting the platform's potential to offer rapid, cost‐effective pre‐screening of clinically relevant mRNA therapeutic delivery vehicles.

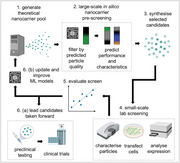

## 1 Introduction

**Figure 1:**
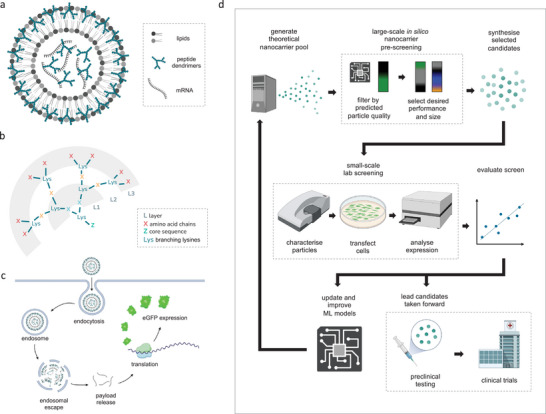
DLN schematics and in silico nanocarrier screening workflow. A) Cartoon schematic of a DLN highlighting individual components. B) Simplified three‐layer peptide‐dendrimer diagram. C) Cartoon visualization of hypothesised cell transfection process via endocytosis. D) Diagram showing key steps of our proposed in silico prescreening and DLN candidate identification workflow. Created with BioRender.

Lipidic nanocarriers are a broad class of lipid‐based vectors with the potential to stably package and deliver emerging nucleic acid therapeutics with high efficiency and precision.^[^ ^1^, ^2^ ^]^ Lipid‐based vectors are generally more biocompatible, and have often shown less immunogenicity, and lower cytotoxicity, compared to viral delivery systems and synthetic polyplexes.^[^ ^3^ ^]^ Two of the most studied lipidic nanocarrier variants for mRNA delivery are solid lipid nanoparticles (LNPs) and lipoplexes (LPXs). LNPs are usually composed of multiple lipids formulated to generate tight complexes with their payload. They have recently attained a high‐profile owing to their use as intramuscular delivery vehicles for SARS‐CoV‐2 vaccination.^[^ ^4^, ^5^ ^]^ LNPs have a tendency to accumulate in the liver when administered intravenously,^[^ ^2^ ^]^ and usually contain a polyethylene glycol (PEG) lipid to help stabilize the particles for prolonged circulation in the body, which can enhance clinical response.^[^ ^6^ ^]^ However, PEG lipids often induce the production of PEG‐antibodies, which can lead to highly negative side effects including anaphylaxis5. Although complete liver de‐targeting has not yet been achieved,^[^ ^7^, ^8^ ^]^ LNPs can be modified to improve non‐hepatic tissue targeting, facilitating applications beyond vaccination.^[^ ^9^, ^10^ ^]^ In contrast to LNPs, LPXs are formed by mixing liposomes (usually containing at least one cationic lipid) with the payload1. This typically leads to the formation of multiple lipid layers around an aqueous centre1. LPXs have been shown to be competent immunotherapy delivery vehicles for treating cancer^[^ ^11^, ^12^ ^]^ and other diseases^[^ ^13^ ^]^ owing to their ability to target the spleen without modification. However, active targeting of other tissues has proven difficult to achieve. Alongside the two prior mentioned lipidic nanocarrier systems, novel hybrid systems have been recently developed, with the hope of overcoming the targeting issues and immunogenic problems associated with LNPs and LPXs while, importantly, improving transfection efficiency. For example, dendrimer lipid nanocarriers (DLNs) are an emerging class of lipidic nanocarriers that incorporate an additional dendrimer component made from either synthetic polymers^[^ ^14^ ^]^ or branched peptides.^[^ ^15^ ^]^ Both classes of DLNs have shown high transfection efficiency, owing their capacity to package and protect the nucleic acid payload more readily than lipid‐only systems.^[^ ^15^, ^16^, ^17^, ^18^ ^]^ Peptide‐dendrimers have the advantage of being intrinsically more biocompatible as they are formed using naturally occurring amino acids.^[^ ^15^ ^]^ They are generated via the strategic placement of lysine residues that permit branching to create a multi‐layer amino acid sequence^[^ ^15^ ^]^ (**Figure** **1**a). We have observed DLNs to have advantages over other lipidic nanocarriers both in vitro and in vivo. For example, our top candidate from prior laboratory screens, which serves as a baseline control peptide‐based DLN in this study, consistently outperforms state‐of‐the‐art LPXs^[^ ^11^, ^12^ ^]^ by fourfold, and LNPs^[^ ^9^ ^]^ by fivefold when deployed to deliver mRNA payloads to human cells (Figure S1A, Supporting Information). Additionally, we have since observed many other DLN formulations to considerably outperform these competing technologies in vitro (Figure S1B, Supporting Information). Encouragingly, in in vivo myeloid/spleen targeting experiments we also found that DLNs outperform state‐of‐the‐art LPXs, are better able to de‐target the liver, and show lower toxicity and immunogenicity (Figure S6, Supporting Information). Like LPXs, DLNs normally have an aqueous core, but the addition of a peptide‐dendrimer can increase particle stability, and facilitates a high degree of control over positional charge‐distributions and hydrophobicity / hydrophilicity.^[^ ^18^, ^19^ ^]^ This can influence tissue distribution, along with both payload packaging and release.^[^ ^18^, ^19^ ^]^ Peptidic DLNs have also been shown to be less cytotoxic than lipid‐only formulations,^[^ ^18^ ^]^ and have the potential for active tissue targeting through conjugation of functional peptide sequences or other chemical modifiers to their peptide‐dendrimer components. This has the potential to overcome the hepatic accumulation seen in both LPXs and LNPs, along with synthetic‐polymer based DLNs.^[^ ^16^ ^]^

Several design rules for optimal peptide‐based DLN formulations have been manually discerned from hypothesis driven research that appear to result in consistently higher transfection efficiency with different payloads.^[^ ^15^, ^20^ ^]^ Peptide‐dendrimers are generally designed to be largely cationic in order to enhance binding efficiency with negatively charged nucleic acids.^[^ ^21^ ^]^ For delivery of DNA, it has been observed that the charge/transfection performance relationship is much more apparent when cationic residues are dispersed uniformly with hydrophobic residues throughout the branched layers of the peptide‐dendrimer, rather than grouped together.^[^ ^15^ ^]^ Research has also shown that DLNs with a greater number of branching layers (increasing from 1 to 3 layers) in their peptide‐dendrimer components correlates with an increase in transfection efficiency with DNA payloads.^[^ ^15^ ^]^ Disrupting this distribution by swapping just a single amino acid position from one to another can lead to a significant change in performance.^[^ ^15^ ^]^ The choice of amine to phosphate (N/P) ratio (the number of cationically charged amine groups in the peptide‐dendrimer to phosphate groups in the nucleic acid payload) appears to have a sequence‐specific optimum, however, the relative change in performance induced by the distribution of cationic and hydrophobic residues has been shown to be somewhat independent of N/P ratio; although is slightly more apparent at higher N/P ratios.^[^ ^15^ ^]^ Despite the general observations outlined above, discovering optimal nanocarrier formulations that result in higher delivery efficiency, while maintaining stable and uniform particle populations, is still a challenging task. Optimal peptide‐dendrimer sequences likely also differ between DNA and RNA payloads.^[^ ^15^, ^20^ ^]^ Moreover, the relationship between component properties and outcomes is not typically linear and previous work investigating different DLN formulations has covered a very small proportion of the potential screening space.

To address design complexity, and to avoid late‐stage failure in the therapeutic pipeline, an important early step in nanocarrier development for clinical applications is to screen diverse libraries for promising formulations in vitro to assess particle quality and payload delivery efficiency.^[^ ^22^, ^23^, ^24^ ^]^ Despite advancements in high‐throughput automation and parallelization, laboratory screening can be costly and time‐consuming owing to the enormity of the screening space.^[^ ^23^, ^24^ ^]^ Moreover, performing a physical grid search of all possible formulations using current high‐throughput screening (HTS) strategies would be virtually impossible even when considering the few routinely combined lipid components for formulating LNPs and LPXs; meaning it is highly probable that many potentially high‐performing formulations are routinely missed. Despite their advantages, DLNs pose a unique screening challenge, as their additional peptide‐dendrimer components add a further layer of complexity relative to conventional lipidic vectors. These branched peptides are constructed using solid phase synthesis, permitting tight control of the number of layers, along with exact sequence and number of residues in each branch.^[^ ^15^ ^]^ This facilitates the design of specific peptide sequences to optimise nucleic acid packaging and delivery,^[^ ^15^ ^]^ but also leads to extremely varied formulation pools with diverse features. Along with near‐infinite component ratios, there are innumerable peptide‐dendrimer sequences that could be incorporated in a DLN. For example, if we only considered the 20 naturally occurring amino acids, and allow no more than four amino acids per branch, with a three amino acid core sequence (excluding branching lysines), an exhaustive search would still require the screening of sixty‐eight quintillion (6.8 × 10^19^) theoretical formulations. Clearly, this is not feasible and necessitates the adoption of a more discerning strategy.

Given the highlighted difficulties of manually predicting particle structure and functional performance of DLNs purely from their quantifiable component properties using a conventional hypothesis driven approach, and the infeasibility of brute force screening, we hypothesised that using machine learning (ML) to pre‐screen potential nanocarriers in silico could facilitate the elimination of poor designs and reduce physical screening to smaller, more promising, candidate pools. If ML models trained on a small fraction of the potential DLN landscape are capable of practically actionable accuracy, this would reduce the volume of physical lab‐based screening considerably. By investigating the relationships between descriptors and outcomes, models could also help to elucidate key features that contribute to nanocarriers' capacities to form well‐packaged, monodisperse populations, and their subsequent mRNA delivery performance. This would contribute to both manual DLN design and mechanistic understanding. Additionally, we suggest that models will continue to improve with access to additional data from the ML‐guided screens, leading to a cycle of ever‐improving in silico pre‐screening performance. Below we describe the implementation of this ML‐guided DLN screening workflow (Figure 1D), and report highly encouraging results including the identification of high performing candidates. To our knowledge, this is the first time ML has been successfully deployed to predict the transfection efficiency of lipidic nanocarriers delivering mRNA to human cells.

## 2 Results

### 2.1 Data Exploration

**Figure 2:**
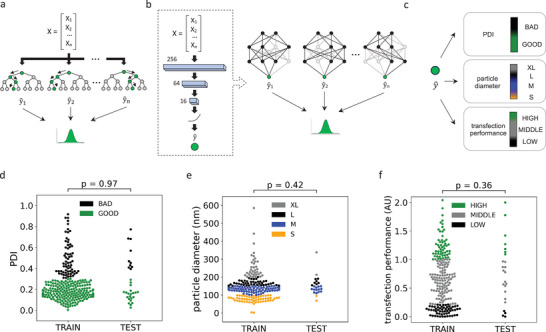
Computational modelling and data processing strategy. A) Diagram of our random forest. Multiple independent trees use random resampling of the dataset and features to make individual predictions. This generates a distribution of outputs that can then be used to estimate model confidence, and aggregated to accurately predict the target of interest. B) Schematic of our three‐layer neural network (right) and visualization of the Monte Carlo dropout approach to prediction. During inference connections in the trained network are disabled at random over multiple iterations, giving a distribution of outputs from individual estimators that can be used to estimate model confidence, and aggregated to obtain an accurate prediction of the target of interest. C) Simplified visualisation of our post hoc classification approach, where aggregated predictions or distributions of individual estimator outputs from our ML regression models can be converted into class labels based on predefined thresholds. D–F) Swarm plots showing the distribution of our targets of interest (PDI, particle diameter (nm), and normalized transfection performance, respectively) in the training and testing datasets, colored by their class labels. P‐Values are from non‐parametric Mann‐Whitney *U* tests.

To investigate, we first acquired a dataset of dynamic light scattering (DLS) measurements and normalised transfection performances (see Experimental Section) associated with over 300 unique DLN formulations. We selected 23 unique DLN features for training our models. These features were selected for their likely biochemical relevance (Table S1, Supporting Information). We aimed to use features that were either known (i.e., the component ratios: N/P ratio and lipid to payload (L/P) ratio), or reliably computable. We avoided features that would require experimental measurement, as this would undermine the aim of the study: to enable in silico screening of theoretical candidates before having to actually formulate them in the laboratory. Peptide‐dendrimer features included the number of branching layers and molecular weight (Da) – indicators of the size and complexity of the molecules. We also used the number of cationically charged residues (lysine, arginine, and histidines), the net charge (*Z*) at various pHs from cytoplasmic (pH 7.4) to that of the mature lysosome (pH 4.5), the isoelectric point (pI) of the entire dendrimer, and the pI of individual layers and the core, and the total number of histidines. These features may be influential on endosomal escape due to varying buffering capacities and conformational changes as the lysosome matures. Moreover, we included features associated with hydrophilicity and hydrophobicity, including proportion of the peptides that were composed of hydrophobic residues and the sum of the Hopp‐Woods scores^[^ ^25^ ^]^ of constituting residues. Both charge and hydropathy features have potential effects on cellular uptake and endosomal escape through alteration of electrostatic interactions. The presence or absence of cysteine was also used, as this factor could lead to dimerisation, altering both particle formation and function. Finally, we computationally estimated the absorbance of the peptide dendrimers at both 205 and 280 nm. Although these are normally proxies for measuring protein concentration, in this case they were utilized to provide a unique numerical signature for the underlying dendrimer sequence. After performing feature extraction and processing, the DLN library was manually analysed for any obvious relationships between extracted features and clinically relevant lab‐based measurements (our targets of interest), including sample polydispersity (PDI), particle diameter (nm), or in vitro transfection performance. First, we examined our continuous features for any apparent linear correlations with our targets. Although some features correlated with each other (e.g., molecular weight and number of layers), no single feature had a strong linear association with any of the three measurements of interest (*R* ^2^ > 0.5), and similarly none of the three target attributes were linearly predictive of each other (Figure S1C–E, Supporting Information). Next, we investigated differences in distributions of the target measurements when partitioned by DLN component ratios using nonparametric Mann‐Whitney *U* tests. We identified very few significant differences in the overall distributions of PDI, particle diameter, and transfection performance between different N/P ratios, and (L/P) ratios (Figure S2, Supporting Information). One notable exception was that on average, particles with N/P ratios less than 1 had smaller diameters (*P* < 0.05) and lower polydispersity (*P* < 0.05). These particles also transfected better (*P* < 0.05). N/P 0.16 also resulted in smaller, less polydisperse, samples than N/P 0.6 formulations, although N/P 0.6 formulations had higher average transfection performance. There was no significant difference in overall PDI distributions at N/P ratios above four. Formulating at L/P 7.5, on average, led to higher transfection performance than L/P 10, but no significant performance differences were detected at other L/P ratios. To investigate further, we defined categorical thresholds for each target of interest and looked for differences in feature distributions in each category (**Figure** **2**D). We defined a “GOOD” particle as having a PDI ⩽0.3, given that this threshold has been defined previously as a clinically acceptable level of polydispersity.^[^ ^26^ ^]^ Size categories were largely defined out of interest based on previous studies demonstrating their approximate association with different uptake mechanisms and cell/tissue specificities.^[^ ^27^, ^28^, ^29^, ^30^, ^31^, ^32^, ^33^, ^34^ ^]^ Particle diameter was divided into four categories: small, “S” (< 100 nm); medium, “M” (100‐150 nm); large, “L” (>150 nm and ⩽200 nm); and extra large,“XL” (> 200 nm). Finally, normalised transfection performance was divided into “LOW”, “MIDDLE”, and “HIGH” performance DLNs based on the interquartile range (IQR) of the full dataset (excluding any samples with a PDI > 0.3). Low performing particles were those with values below Q1, middle particles were those falling inside the IQR, and high performing DLNs were any with performance falling above Q3. Although there was a great degree of overlap, many statistically significant differences between feature distributions and target categories were uncovered (Figure S3, Supporting Information). Good quality DLN samples were generally associated with lower N/P ratios. More cationically charged residues in the peptide‐dendrimer components also more often resulted in samples with lower PDI. Although slightly higher transfection efficiency was associated with lower N/P, unlike PDI, fewer charged residues often led to better transfection, indicating that features leading to a high quality particle do not necessarily lead to better performance. Taken together, the above analyses demonstrated that although associations existed between features and targets, they are not linearly deducible, and substantial overlap exists between DLN quality, size, and performance distributions for all features used in this study. Hence, manually selecting a specific component ratio or any combination of the 21 peptide‐dendrimer features described in Table S1 (Supporting Information) was no guarantee of a high quality, high‐performing DLN. Nonetheless, the statistically significant differences observed were encouraging, as they demonstrated that the selected features had some strong association with the measurements of interest and may therefore be useful for computational modelling.

### 2.2 Computational Modelling

Following feature exploration, we constructed several ML regression models designed to predict our targets of interest using advanced non‐linear methodologies. We chose to use regression, rather than sorting DLNs into the above outlined categories directly using classification models. This was because: i) we did not want to discard useful information, ii) we were interested in ranking the specific performance of DLNs, and iii) the possibility for classification was retained as classes can simply be assigned to predicted values post hoc (Figure 2C). All samples in the initial dataset were leveraged when training the PDI prediction model (including the “BAD” examples) so that PDIs > 0.3 could be readily predicted. In contrast, the particle diameter and in vitro performance models were trained only on “GOOD” particle data (i.e., that had a lab‐measured PDI ⩽ 0.3), as the observed size and performance of heterodisperse or precipitated samples are unreliable and may confound computational modelling; this excluded around 24% of the initial dataset. To evaluate the models, and to avoid overfitting to either the training or validation sets during model selection and training, we used the standard best‐practice of randomly splitting the initial DLN library dataset into two cohorts: a training set comprising approximately 90% of the available data, and an independent testing set comprising the remaining 10%. The datasets were different for the PDI model relative to the particle diameter and transfection performance models, given that high‐PDI samples had first been filtered from the latter sets. Mann‐Whitney *U* tests showed no significant difference between the training and hold‐out test set distributions for any of the three targets, indicating that these random subsets were representative of the full dataset (Figure 2D). The test sets were held‐out throughout the entire model selection and training pipeline and only deployed at the final step for evaluation purposes. To predict PDI and particle diameter (nm) we trained random forest (RF) regressor ensembles.^[^ ^35^ ^]^ RFs are powerful, widely used, and, unlike neural networks, generally perform well on tabular data out‐of‐the‐box without requiring exhaustive architectural optimization. It is also possible to estimate uncertainty by analysing the variance between predictions made by individual decision trees in the forest (Figure 2A). The only caveat of using tree‐based ML approaches is that they generally struggle to extrapolate beyond the range of observations that appear in their training sets. As PDI is limited to values between 0 and 1, and both PDI and particle diameter only needed to meet a specific threshold for clinical use, this limitation of RFs was not an issue in such cases. However, transfection performance is unbounded and no upper limit is considered detrimental. Hence, in this case a model capable of both extrapolation and complex non‐linear function approximation, such as a neural network, was deemed more suitable. Typically, trained neural networks output single values when new examples are passed to the model for inference. This makes obtaining the degree of confidence over individual predictions difficult to retrieve. We overcame this limitation by using an approach called Monte Carlo dropout,^[^ ^36^ ^]^ where connections in the trained network are disabled at random prior to predicting DLN performance. After multiple iterations of this, the predictions are aggregated and the mean taken as the final result (Figure 2B). This permits the estimation of confidence in any prediction by analysing the variance across Monte Carlo iterations. Compared to other ensembling approaches such as bagging,^[^ ^35^ ^]^ this strategy is less computationally expensive as it does not require any retraining. Results are also improved by effectively ensembling multiple models together. Using ensemble models also has further advantages. For example, post hoc classification ‐ discussed earlier ‐ can be performed in two different ways when using ensembles of estimators: i) the average predicted value from all estimators can be assigned to its corresponding class, or ii) each individual estimator's prediction can be assigned its own class and the proportion of estimators assigned to each class taken as the predicted probability of that particular class. The former approach is simpler, but retains less information about the uncertainty of the model when the post hoc classification is performed.

### 2.3 Model Evaluation

**Figure 3:**
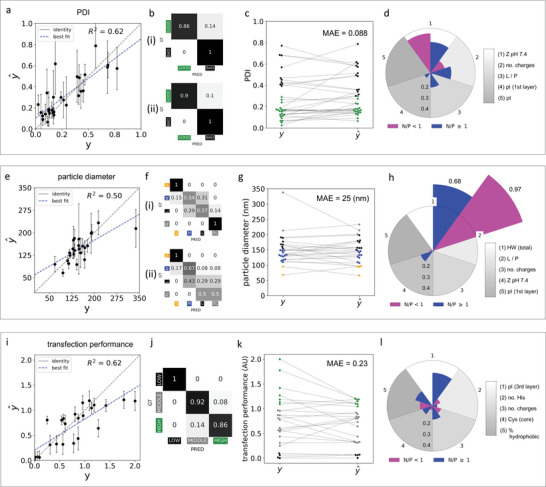
Model evaluation and feature importance analysis. A, E, I). Scatter plots showing the correlation between true *y* and predicted $\hat{y}$ values for targets of interest when models are deployed on the hold‐out test set. Error bars depict the standard deviations across estimators in the ML models for each individual prediction. The overall *R* ^2^ is also given for each set of predictions. B, F, J) Confusion matrices for post hoc classification on the test set using (i) direct translation of the mean prediction into the respective class, or (ii) translation of each individual estimator in the ensemble into its associated class and the largest proportion (majority vote) used as the final class assignment. For (J) transfection performance, one matrix is shown as results were identical. GT = ground truth, PRED = predicted; matrices are normalised by row (true label). C, G, K) Swarm plots showing true *y* test set values and model predicted $\hat{y}$ values for targets of interest with lines matching each true value to its corresponding predicted value. The MAE is also reported. D, H, L) Polar bar plots showing the top five most important features for predicting associated targets of interest obtained by performing permutation importance analysis with the corresponding ML models. Shown are the importance of these features at N/P ratios below 1 (magenta) or above 1 (blue). Feature importances (increase in MAE) have been scaled between 0 and 1 in order to better visualize their proportional contributions (see Experimental Section).

First, we examined the raw outputs of our regression models when deployed on the independent test set and compared these to ground truth measurements obtained in the laboratory. Models for all targets showed strong positive correlations with true values, and this was particularly evident for both PDI and transfection efficiency (**Figure** **3**A, E, I). Post hoc classification using both approaches outlined above gave good results for PDI (*F* ^1^ = 0.92 and 0.95, respectively) and transfection performance (for both post hoc methods *F* ^1^ = 0.93). For PDI, both classification strategies gave 100% precision with zero “BAD” samples being classified as “GOOD”; meaning the model was able to filter out all poor quality samples. However, approach (ii) resulted in slightly higher recall of “GOOD” particles (Figure 3B). For classification of transfection performance the mean precision and recall across all three classes was 93% (Figure 3J). Classification of “LOW” performing DLNs gave perfect precision and recall (100%), with classification error increasing slightly for “MIDDLE” (precision and recall 92%) and “HIGH” performing particles (both precision and recall 86%). This demonstrated that the model was, at worst, able to filter out DLNs that are unlikely to transfect well. For particle diameter, post hoc classification was slightly less effective (*F* ^1^ = 0.62 and 0.47, for post hoc approaches (i) or (ii), respectively), with the model often mistaking medium particles for large, and vice versa (Figure 3F). Although, with a mean absolute error (MAE) of 25 nm (Figure 3G), the model was still highly effective at approximating actual particle size, and confusion could be expected in these class bands given their resolution was just 50 nm apart. To solidify results and further assess generalization, we randomly divided our dataset into three additional train‐test splits and assessed the regression performance of all of our models across these. All models maintained an average *R* ^2^ > 0.5, indicating a strong relationship with the ground truth values across the three random splits (Figure S7, Supporting Information); MAEs were also similar to the results obtained in the original evaluation (PDI: 0.059 ± 0.01; particle diameter (nm): 26 ± 3; transfection performance: 0.22 ± 0.02). Overall, evaluation of our ML models using an independent hold‐out test set showed strong performance both in direct regression, and post hoc classification. We, therefore, decided that our models were suitable for conducting a large‐scale in silico DLN prescreen trial with the aim of discovering high quality, high performing, candidates.

### 2.4 Feature Importance Analysis

To uncover which features were most influential in predicting our targets of interest, we performed permutation importance analyses to rank input features by contribution (Table S2, Supporting Information). For all models, N/P ratio was observed to be the greatest contributor; N/P ratio contributed 65% to the PDI model, 72% to the particle diameter model, and 52% to the transfection performance model. Despite these large contributions, the influence was not linear (Figure S1D‐E, Supporting Information) and 28–48% must be explained by other input features. We also observed that the influence of N/P ratio was highly dependent on other features, as different features had very different influences depending on the N/P ratio. Figure 3D, H, I, show the five top ranked features for each model using this approach, excluding N/P ratio. The contribution of these five features at all N/P ratios above 1 (blue) or below 1 (magenta) is also visualized, in order to show how differences in N/P ratio appear to interact with other attributes of the nanocarrier. One initial observation was that the PDI and particle diameter models appeared to share four out of five top features with each other, but only one top feature was shared between these and the transfection performance model, reinforcing observations that particle formation is influenced by different factors compared to particle function. For PDI, the influence of different features was somewhat similar when DLNs were grouped by N/P ratios above or below 1. The two most important factors were the net charge of the peptide‐dendrimer component at pH 7.4 and the total number of cationically charged residues. On average, “GOOD” samples had higher net charges at pH 7.4 (17.87 vs. 14.86) and more cationic residues (18 vs. 15) than “BAD” samples. As the nucleic acid payload is negatively charged, this likely indicates the importance of charge interactions for payload packaging when particles are formulated in neutral pH buffers; with poor packaging leading to a less uniform distribution of lipid‐dendrimer‐mRNA structures in the sample. Interestingly, particle diameter for DLNs with N/P ratios below 1 was almost entirely influenced by L/P ratio (97%), indicating that the lipid component is responsible for the overall size of a particle when few peptide‐dendrimer particles are present. Generally, lower L/P ratios led to larger particles. However, interestingly, the smallest particles with NP ratios below 1 were in class “M”, with no small “S” particles observed. This indicates that a significant amount of peptide‐dendrimer component is required to produce particles with diameters < 100nm, likely owing to their capacity to form compact complexes with the mRNA payload. This discovery was further supported by the observation that L/P ratio had very little effect on the size of DLNs with N/P ratios above 1, which was, in contrast, greatly influenced by the total Hopp‐Woods score (68%) – a proxy for the overall hydrophilicity of the peptide‐dendrimer component^[^ ^25^ ^]^ –, and the net charge at pH 7.4 (13%). Above an N/P ratio of 1, on average, smaller particles had more hydrophilic peptide‐dendrimers than larger particles (Hopp‐Woods totals: “S”: 37.84, “M”: 31.2, “L”: 26.96, and “XL”: 21.98), which also had a greater net charge (*Z*: “S”: 21.2, “M”: 19.14, “L”: 16.75, and “XL”: 12.87). Increased hydrophilicity may influence how the peptide‐dendrimer is arranged throughout the lipidic structure of the nanocarrier leading to more densely packaged particles, and more tightly bound peptide‐dendrimer‐payload complexes. For transfection performance, at N/P ratios above 1, the isoelectric point of the 3^*rd*^ layer of the peptide‐dendrimer was highly influential. Combined with the observation that the second and third most important features were the number of histidine residues in the peptide‐dendrimer sequence and the number of cationically charged residues, this perhaps indicates that, following cellular uptake, changes in charge during the acidification of the endosome is important for endosomal escape and subsequent payload release into the cytoplasm during lysosomal maturation (Figure 1C). Further supporting this hypothesis, we observed that “HIGH” performing DLNs in the dataset had, on average, 2.5 times more histidine residues in their peptide‐dendrimer components than “LOW” performers. Histidine's low basicity may permit enhanced buffering capacity as the pH of the endosome/lysosome decreases.^[^ ^37^ ^]^ The importance of the 3^*rd*^ layer specifically may indicate different nanocarrier packaging behaviors for different sized peptide‐dendrimers in DLNs formulated at divergent N/P ratios, leading to different particle conformations. Only 34% of “HIGH” performing DLNs in the dataset had a 3^*rd*^ layer, compared to 76% of “LOW” performing nanocarriers. Where a 3^*rd*^ layer was present, its pI in “HIGH” performers was on average 1 pH lower at pH 9.7, whereas for “LOW” performers it was pH 10.8. This may indicate greater stability and less aggregation at lower pH in high performing candidates, allowing for smoother endosomal escape.

### 2.5 Candidate Identification via In Silico Prescreening

**Figure 4:**
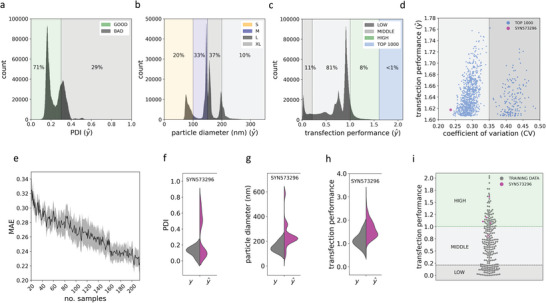
Large‐scale in silico screening and candidate identification. A–C) Histograms showing the predicted values $\hat{y}$ for associated targets of interest across the computationally generated DLN formulation library. The predicted PDI histogram depicts the entire ≈ 4.5 million candidate pool. The particle diameter and transfection performance histograms depict only the filtered (predicted PDI ⩽ 0.3) sub‐library (≈ 3.25 million formulations). Class bins and the percentage of DLNs falling into them are also reported. D) Scatter plot showing the top 1000 DLN candidates with the highest predicted transfection performance. Predicted transfection performance is shown on the *y*‐axis and the coefficient of variation (CV) for each prediction is shown on the *x*‐axis. E) Plot showing the reduction in mean absolute error (MAE) for normalised transfection performance on the validation set, when the neutral network is trained on 20 to 200+ examples. A new fixed held‐out set consisting of 10% of all available data was used for validation. Error margins are standard errors over ten experiments; the train‐validation split for each was selected using bootstrapping with replacement. F–H) Split violin plots showing kernel density estimations for all replicate measured values *y* and for all individual ML estimator predictions $\hat{y}$ for candidate SYN573296. I) Swarm plot showing the laboratory measured transfection performance of our entire dataset (PDI ⩽ 0.3) with results of all individual laboratory replicates for candidate SYN573296 superimposed on top.

We hypothesised that performing large‐scale predictions on millions of potential DLN formulations using our ML models as an in silico pre‐screening platform would allow us to reduce our physical screening to just a handful of leads, enabling the identification of novel high performing DLNs while minimizing physical resources. We generated a library of over 4.5 million unique DLN formulations by randomly recombining different sequence motifs of existing peptide‐dendrimers at multiple component ratios (see Experimental Section). We used this approach over entirely random de novo sequence and component ratio generation, primarily to ensure that our theoretical formulations' features did not deviate too much from the DLN library used to train the models. This way we could have some degree of confidence in any predictions made. As shown in Figure 1D, these theoretical formulations were first passed to our particle quality model where DLNs that were predicted to result in poor quality samples (“BAD” PDI) were filtered out. This eliminated 21% (around 1.3 million designs), leaving around 71% of the in silico library intact (**Figure** **4**A). This was in line with the 24% of examples classified as “BAD” in the initial library used to train the model. Next, we passed the filtered library, now containing around 3.25 million formulations to our particle diameter prediction model. Interestingly, the predicted sizes for the filtered in silico library appeared to map relatively well to the predetermined size band categories when plotted as a histogram, with distinct peaks visible in the small, medium and large bands (Figure 4B). However, there was some overlap, especially between the large and extra large size categories. Although we were interested in the size of our in silico candidates, in this case we did not filter out any particular size banding. This was primarily because we had slightly less confidence in the particle diameter model, given its results on the independent test set. Hence, all 3.25 million remaining formulations were passed forward to our transfection performance neural network. To select a top candidate for real‐world synthesis, we decided to implement a precautionary confidence/performance trade‐off strategy. We first identified the top 1000 DLN formulations with highest predicted transfection performance (Figure 4C). Next, we organized these by their coefficients of variation (CV) (Figure 4D), with lower CVs indicating the higher levels of agreement between different Monte Carlo dropout estimators, and, hence, higher model confidence in the predictions made. Interestingly, this revealed two very distinct, highly separated, groups within the top 1000: a high CV group, and a low CV group. To understand this grouping, we looked at the average min‐max scaled feature values for each group (Figure S5D, Supporting Information), which showed that high CV (low confidence) predictions were, on average, associated with DLNs containing larger peptide‐dendrimers with more layers, and more hydrophobic residues. This suggests that as the size of the dendrimer increases along with its amino acid sequence complexity, reliable prediction of performance becomes more difficult. The top most confident design, SYN573296, from the top 1000 predicted best performers, was selected to be synthesised in the laboratory. The specific scaled feature values for this candidate are also shown in (Figure S5D, Supporting Information).

### 2.6 Candidate Evaluation

The predicted novel peptide‐dendrimer component for nanocarrier candidate SYN573296 was synthesised using solid‐phase synthesis, with DLNs then formulated at the best predicted component ratios: N/P ratio 8 and L/P ratio 7.5. We formulated five independent replicate samples for the candidate and tested each replicate by DLS, with each well‐formed sample (PDI ⩽ 0.3) taken forward for in vitro transfection performance testing. After candidate DLNs were physically characterized using DLS, results were compared to predicted values. Figure 4F,G show split violin plots for PDI and particle diameter, representing the kernel density estimations of replicate laboratory formulations versus all 512 individual tree predictions from the RF models for these targets. Candidate SYN573296 consistently resulted in extremely good quality samples with PDI < 0.2, as predicted. For particle diameter, the model had predicted that the SYN573296 was likely to be “XL” (> 200 nm), although when measured by DLS candidates had a mean size of 154 nm, for SYN573296 (the smaller end of the “L” class). The candidate was predicted to fall into the “HIGH” performance category for transfection. Fig 4H, shows violin plots for lab measured transfection performance versus all neural network estimators. Corresponding to predictions 80% (4 out of 5) of SYN573296 replicates showed high performance in in vitro cell transfection experiments, consistently falling in the “HIGH” category. Figure S5A– C (Supporting Information) shows the results of post hoc classification, where the proportion of estimators predicting each category is reported. The CV of transfection performance for candidate SYN573296 across replicates was 0.25; compared to a predicted CV of 0.24 across the individual neural network estimator predictions. This indicates that although high biological and experimental variation may contribute to prediction inaccuracies, the model reported a similar expected variation. Overall, the identification of SYN573296, a high performing, high quality, DLN formulation, through screening of a single top candidate was extremely encouraging. Furthermore, we observed that our transfection model tends to increase its performance linearly based on the quantity of available data (Figure 4E) from 20 to over 200 examples, demonstrating that in silico pre‐screens will likely continue to improve as more training data is acquired and fed back to our models.

## 3 Discussion

Taken together, these experiments show that our ML models have practically applicable real‐world performance and can help identify nanocarrier candidates with clinical potential. We were able to pre‐screen over 4.5 million candidate nanocarriers in silico; a quantity that would have required almost 12,000 individual 384‐well plates per step (assuming no technical or experimental replicates) based on advanced robotics‐based HTS approaches.^[^ ^23^ ^]^ In contrast, by selecting and synthesising just the top formulation from this screen, we were able to obtain a consistently high quality, high performing nanocarrier. Furthermore, the construction of our computational screening platform required a small initial dataset of just ≈ 300 formulations. We also demonstrated that our system continues to improve with additional data, meaning that each mini laboratory screen improves the pre‐screening platform further, by providing its ML models with new information. Feature analysis demonstrated that at greater N/P ratios, on average nanocarriers producing high quality, monodisperse populations had a higher net charge and a higher proportion of cationic residues in their dendrimer components. This likely leads to more efficient, denser, packaging of the negatively charged nucleic acid payload, forming a complex that is more resilient to perturbation and degradation. In addition, we discovered that “HIGH” performing DLNs in the dataset had, on average, 2.5 times more histidine residues in their peptide‐dendrimer components than “LOW” performers. Histidine has a low basicity and enhanced buffering capacity at lower pH values, perhaps protecting the payload during lysosomal maturation, providing more capacity for endosomal escape. It was also apparent that in nanocarriers with three‐layer dendrimers, the 3^*rd*^ layer, had a lower pI, on average. Similarly to the presence of histidines, a lower pI in these large multi‐branched constructs may allow for more efficient payload release as the endosome acidifies by reducing electrostatic interactions between the payload and cationic peptide‐dendrimers. Although the attributes we were able to predict in this study provide an encouraging proof‐of‐principle, it is important that future work also focuses on other key areas with relevance to clinical application, including predicting in vivo biodistribution, tissue targeting, and nanocarrier toxicity to pre‐assess safety. This will necessitate innovative experimental design strategies that minimize the number of animals required, as accurate ML models often need many training examples. We also hypothesise that, as more data is gathered from more diverse DLN formulations, novel features in conjunction with more complex feature representations may be required to predict complex characteristics. We previously emphasised our preference to use features that could be calculated entirely computationally without having to synthesise potential candidates. However, now we have shown that certain characteristics can be predicted computationally with high accuracy, it may subsequently be possible to use such ML predicted aspects themselves as input features for new ML models. Notably, in addition to the core work reported in the paper, we subsequently created a RF model capable of accurately predicting the zeta potential of nanocarriers (*R* ^2^ = 0.72 ± 0.01 and MAE = 2.75 ± 0.34 mV) from just 89 of the examples in our dataset (Figure S7D, Supporting Information). Given that zeta potential is a measure of the surface charge and stability of particles in a colloidal dispersion, using the predicted zeta potential value, along with the predicted particle diameter of a nanocarrier, could be a useful input to a model designed to predict therapeutic delivery and efficacy. Past studies have shown that both size and zeta potential are significant factors contributing to particle stability, cellular uptake, and payload release.^[^ ^38^ ^]^ Overall, we propose that similar approaches might also be applicable more broadly to optimise a variety of mRNA delivery systems such as LNPs, LPXs and synthetic DLNs; where features such as lipid species, and molar ratios could be used as input features to a model. The application of ML approaches for pre‐screening high‐quality samples from in silico design pools has significant potential to reduce both the cost and time required to bring mRNA therapies to the clinic.

## 4 Experimental Section

### Nanocarrier Formulation

*—DLN Formulation*

To formulate DLNs, the peptide‐dendrimers were prepared in solutions of 10 mgml^−1^ in water or 0.5 mgml^−1^ in water. The final volume was 65 µl with an mRNA payload concentration of 50 µgml‐1. Tube A (peptide‐dendrimer): to a sterile 1.5 ml polypropylene tube, 50 mM HEPES buffer (3.25 µl), sterile water (to give a final volume of 6.50 µl) and 10 mgml^−1^ peptide‐dendrimer stock solution (7.879 × 10^−5^ mmoles N) was added for and N/P ratio of 8. For an N/P ratio of 0.15625, a 0.5 mgml^−1^ peptide‐dendrimer stock solution (1.539 × 10^−6^ mmoles N) was used. The quantities of peptide‐dendrimer stock solution and water vary depending on the molecular weight and charge of the peptide‐dendrimer. Tube B (200 µgml^−1^ mRNA in 25 mM HEPES buffer): to a sterile 1.5 ml polypropylene tube, 200 mM HEPES buffer (134.1 µl), sterile water (724.00 µl) and 1 mgml^−1^ mRNA stock solution (214.50 µl) were added, and the tube was mixed with gentle shaking. Tube C (769 µgml^−1^ lipofectin in 25 mM HEPES buffer): to a sterile 15 ml polypropylene falcon tube, 200 mM HEPES buffer (348.60 µl), sterile water (294.9 µl) and 1 mgml^−1^ Lipofectin solution (2.145 ml) were added, and the tube was mixed with gentle shaking. Mixing: 200 µgml^−1^ mRNA solution (16.26 µl, 3.252 µg, 9.855 × 10^−6^ mmoles P) was mixed into tube A by rapidly pipetting up and down 10 times. This was allowed to sit for 5 min. 769 µgml^−1^ lipofectin solution (42.20 µl, 32.46 µg) was mixed into tube A by rapidly pipetting up and down ten times. The eGFP mRNA used in this study was purchased from Trilink (CleanCap EGFP mRNA (5moU) ‐ (L‐7201)). DOTMA:DOPE, 1:1 (w/w) were obtained from Invitrogen (Lipofectin Transfection Reagent) (Fisher ‐ 18292037).

A variety of DLN formulations were included in the training dataset. These were formulated using a process as described below for an exemplary peptide of the training dataset.

*Example peptide x*: RHCG1‐R or (R)2KRHC‐NH2.

*Example formulations*:

DLN example 1. Example peptide x, N/P ratio 8 (1538 gmol^−1^, 5 charges per peptide‐dendrimer): Tube A (peptide‐dendrimer): to a sterile 1.5 ml polypropylene tube, 25 mM HEPES buffer (3.25 µl), sterile water (0.826 µl) and 10 mgml^−1^ peptide‐dendrimer stock solution (2.424 µl, 24.2 µg, 1.58 × 10^−5^ mmoles peptide‐dendrimer 7.88 × 10^−5^ mmoles N) was added.

DLN example 2. Example peptide x, N/P ratio 0.15625 (1538 gmol^−1^, 5 charges per peptide‐dendrimer): Tube A (peptide‐dendrimer): to a sterile 1.5 ml polypropylene tube, 25 mM HEPES buffer (3.25 µl), sterile water (0.826 µl) and 0.5 mgml^−1^ peptide‐dendrimer stock solution (0.948 µl, 0.474 µg, 3.08 × 10^−5^ mmoles peptide‐dendrimer, 1.54 × 10^−6^ mmoles N) was added.

*LNP Formulation*

Making lipids stock: 135.8 µl DLin‐MC3‐DMA at 39.20 mgml^−1^ in Ethanol, 167.8 µl DSPC at 7.80 mgml^−1^ in Ethanol, 192.8 µl Cholesterol at 12.80 mgml^−1^ in Ethanol, 44.2 µl DMG‐PEG2000 at 14.10 mgml^−1^ in Ethanol and 209.4 µl Ethanol were added to a glass HPLC vial and stored at ‐20°C. This corresponds to a molar ratio of DLin‐MC3‐DMA (50.0%); DSPC (10.0%); Cholesterol (38.5%); DMG‐PEG2000 (1.50%). To tube 1, 9.00 µl of eGFP mRNA at 1.00 mgml^−1^ (9.00 µg), 11.25 µl of sterile water and 2.25 µl of 100mM Citrate buffer (pH 3.0) was added. Final citrate buffer concentration was 10mM. Mixing: 7.50 µl of the LNP lipids stock described above was added into the tube of mRNA, using the pipette to rapidly mix the two solutions until homogenous. DLS results for LNP were: size: 154nm, PDI: 0.183, Zeta potential: +26.4mV. Formulation was based on Cheng et al.^[^ ^9^ ^]^

*LPX Formulation*

42.0 µl of rRNA‐LPX encapsulating eGFP mRNA (L‐7201 TriLink) at a concentration of 50 µgml^−1^. Final buffer composition was 150mM NaCl. To tube 1, 2.205 µl of eGFP mRNA at 1.00 mgml^−1^ (2.205 µg), 7.17 µl of sterile water and 1.65 µl of 4M NaCl was added. To tube 2, 1.746 µl of DOTMA/DOPE liposome (1:1 molar ratio) at 3.50 mgml^−1^ (6.174 µg) and 31.33 µl of sterile water was added. Mixing: 31.50 µl of the liposome solution (tube 2) was rapidly mixed into the tube of mRNA by pipetting up and down rapidly. DLS results for LPX were: size: 182nm, PDI 0.050. LPX formulation was based on Kranz et al.^[^ ^11^, ^12^ ^]^

### Nanocarrier Characterization

The DLN's sizes in nm and polydispersity index (PDI, also labeled as “PdI”) were measured on a ZetaSizer Pro (Malvern Panalytical). The hydrodynamic size was measured using the dynamic light scattering (DLS) technique using the Zetasizer Advance Series – Pro (Malvern Panalytical Ltd, Malvern, UK) according to the manufacturer's instructions. DLS was a very sensitive, non‐invasive method to measure size and size distribution of nanoparticles in a liquid. The Brownian motion of nanoparticles in suspension resulted in laser light to be scattered at different intensities. Analysing these intensity fluctuations allowed to calculate the velocity of the Brownian motion. The size of the nanoparticles could be determined by using the Stokes‐Einstein relationship. With the latest technology, it could measure nanoparticles smaller than 1nm. The data obtained with DLS measurements could also be used to calculate the PDI of particles in solution. PDI was used to estimate the average uniformity of a particle solution. The method of cumulants was a standard technique for analysing DLS data on sample polydispersity. PDI was a number calculated from a 2 parameter fit to the correlation data (the cumulants analysis). The cumulants analysis was used to evaluate the autocorrelation function generated by a DLS experiment. The calculation was defined in ISO 13321 and ISO 22412. PDI values greater than 0.7 indicate that the sample had a very broad size distribution and was not suitable for the DLS technique. The calculations for these parameters were defined in the ISO standard document 13321:1996 E and ISO 22412:2008. To measure the size and PDI, the samples were diluted 8× in 25 mM HEPES buffer (10 µl sample + 70 µl buffer). Parameters: reference material: polystyrene latex, dispersant: water, 25 °C.

### Measurement of Zeta Potential

Zeta potential was a measurement of the magnitude of the electrostatic or charge repulsion/attraction between particles. This could be determined by analysing particle mobility and charge (Zeta potential) using the Electrophoretic Light Scattering (ELS) technique. To measure the zeta potential, the samples were diluted 47× or 70× in 25 mM HEPES buffer (15 µl sample + 685 µl buffer for 47×, or 10 µl sample + 690 µl buffer for 70×) and added to a clean DTS1070 cell. Parameters: reference material: polystyrene latex, dispersant: water, 25 °C. The measurements of the zeta potential were carried out using the Zetasizer Advance Series – Pro (Malvern Panalytical Ltd, Malvern, UK) according to the manufacturer's instructions. The zeta potential was reported in units of millivolts (mV), which represent the magnitude of the electric potential at the shear plane of a particle in the colloidal dispersion.

### Cell Lines and Transfection

HeLa cells (ATCC: CRM‐CCL‐2) were maintained in MEM with 10% (v/v) FBS and 2mM L‐glutamine. HEK‐293 (ATCC: CRL‐1573) were maintained in MEM with 10% (v/v) FBS and 2mM L‐glutamine. A549 (ECACC: 86012804) were maintained in Ham's F12K media with 10% (v/v) FBS and 2mM L‐glutamine. All cells were incubated in a humidified atmosphere in 5% CO2 and 37 °C. 24 h before transfection, cells were seeded in 96 well plates in order to reach 80% confluence. DLN transfection complexes were then overlaid to the cells in full growth medium. The cells were harvested for reporter gene assay 24 h post transfection. Six technical replicate wells were used for each DLN. HeLa were discarded after 20 passages.

### Assessment of Nanocarrier Transfection Performance

The cells were washed twice with PBS and incubated with 50 µl of 1× M‐PER lysis buffer (Thermo 11874111). Plates were protected from light and gently agitated at RT for 15 min to aid cell lysis. 40 µl of lysate from each well was transferred to all black 96 well plates for quantification of eGFP relative fluorescence unit (RFU), absorbance at 535nm, using Molecular devices SpectraMax iD5. The protein content of each cell lysate was determined by mixing the lysate (25 µL) with Pierce BCA Protein Assay Kit (200 µl, Thermo Scientific). After 30 min of 37°C incubation in the dark, absorbance was measured at 562nm with Molecular devices SpectraMax iD5 and converted to protein concentration using a BSA standard curve. RFU/mg of protein represented eGFP expression. For each nanocarrier tested, the mean of the six replicates (minus buffer only background) was taken as the final value. To normalize results between different plates and experiments, the RFU/mg from each DLN‐eGFP transfection was divided by the RFU/mg of a high performing positive control DLN formulation optimized from previous work (Figure S1A, Supporting Information). This gives a ratio (the normalized transfection performance, reported as “transfection performance” in the main text) for each DLN formulation. As an additional quality control, two other positive control formulations with differing levels of performance to the primary positive control were always included in each plate, with data discarded if the ranking of all three controls was inconsistent with previous observations. Figure S8 (Supporting Information) shows the comparative performance of these positive control DLNs across replicate experiments in three different human cell lines: HeLa (cervical), HEK‐293 (kidney) and A549 (lung). For HEK‐293 and A549 six biological replicates was used; for HeLa over 50 biological replicates were used for each DLN. HeLa was chosen as the experimental cell line for ML work, as it showed consistently higher reporter expression overall (RFU/mg protein) than the other cell lines.

### In Vivo Experiments

Wild type mice (n = 3) were intravenously injected with the DLN and mRNA or with myeloid cell targeting lipoplexes (LPX) and mRNA. The mRNA used expresses luciferase (Trilink CleanCapFLuc mRNA (5moU)). For biodistribution experiments, the dose was at 0.75mgkg^−1^ and the luciferase protein expression was measured 6 h post administration. For liver toxicity and cytokine data mice (n = 3) were intravenously injected with LPX and mRNA or the nanocarriers and mRNA (DLN) at 1mgkg^−1^. Plasma was collected 6h post injection to measure various serum parameters. Aspartate aminotransferase (AST) correlates with liver toxicity. IL‐6 and IL‐12 levels indicate immune activation. LPX from Sahin et al. and Kranz et al.^[^ ^11^, ^12^ ^]^ had a proven safety record and was used by BioNTech across their cancer vaccine phase I and II clinical trials. Female CD‐1 or BALB/c mice (6‐8 weeks) were purchased from Charles Rivers. All procedures to minimize suffering during the experiments, the methods of sacrifice, sample collection, anaesthesia, housing, sustenance, and storage conditions were performed under the approval of the Home Office and meet all current regulations and standards of the UK (The Animals (Scientific Procedures) Act 1986). No adverse effects were observed during the study. Animals were housed and maintained in rooms under controlled conditions of temperature (19–23 °C) and humidity (55% ± 10%), photoperiod (12h light/12h dark) and air exchange, with food and water provided ad libitum. The facilities had been approved by the Home Office and meet all current regulations and standards of the UK (The Animals (Scientific Procedures) Act 1986).

### Data Preprocessing and Feature Extraction

All data used in this study was generated de novo. The total dataset size comprised 310 unique DLN formulations. All DLN data was used in feature exploration and modelling of PDI. 234 nanocarriers had PDI ⩽ 0.3; these were used in feature exploration and modelling of particle diameter and normalized transfection performance. Results from four lipid only formulations at L/P ratios of 5, 7.5, 10, and 12.5 were also added to each training set to provide a no‐peptide‐dendrimer particle baseline for the model. A selection of 23 DLN features were used for training models. The individual features and their calculations are described in Table S1 (Supporting Information). Features were min‐max scaled using:

(1) $$x_{scaled}=\frac{x-x_{min}}{x_{max}-x_{min}}$$

Minimum and maximum scaling values are shown in (Table S1, Supporting Information). Maximum values for peptide‐dendrimer sequence‐based features were theoretical maximums based on the inclusion of any natural amino acid and a maximum number of 55 residues. This permits the same scaling values to be used in future experiments, and for novel peptide‐dendrimer sequences. Binary features (e.g., presence of cysteine in the peptide‐dendrimer core sequence) were represented as 0 or 1. Missing features (e.g., pI of the 3^*rd*^ layer in a 1 or 2‐layer peptide‐dendrimer) were represented as ‐1. Hydrophilicity features were computed using values from Hopp and Woods (1981) scale^[^ ^25^ ^]^ and computational estimation of specific peptide‐dendrimer absorbances at 205 and 280 nm was conducted based on Anthis and Clore (2013)^[^ ^39^ ^]^ and Gill and von Hippel (1989),^[^ ^40^ ^]^ respectively.

### Random Forest Modelling

The RF model was composed of 512 estimators (trees). Key hyperparameters were: minimum samples per node split = 2, minimum samples per leaf = 1. No maximum depth was assigned, so decision trees could expand as required without limits. The mean squared error (MSE) was the criterion (loss function) for training the model:

(2) $$MSE=\frac{1}{n}\sum_{i=1}^{n}{\left(y_{i}-{\hat{y}}_{i}\right)}^{2}$$

where *n* is the number of observations, *y*_*i*_ is the target value, and ${\hat{y}}_{i}$ is the corresponding predicted value. The random forest model was built and deployed in Python 3 using scikit‐learn v. 1.2.2.^[^ ^41^ ^]^

### Artificial Neural Networks

To discover an effective neural network architectural configuration a grid search of three‐layer architectures using any combination of 16, 32, 64, 128 or 256 neurons was performed. Each model in the grid pool was validated using threefold cross validation Each abstraction layer in the network was followed by a rectified linear unit (ReLU) activation function and was regularized during training using 50% dropout. A single output node was used to predict the normalized transfection performance. As this target attribute was comprised of unsigned and unbounded values, a softplus activation function was utilized after the output node, where:

(3) Softplus(x)=log(1+exp(x))

The ANN's trainable weights were initialised with random values from −*c* to *c* using a Glorot Uniform initialization function:^[^ ^42^ ^]^

(4) $$c=\sqrt{\frac{6}{n_{in}+n_{out}}}$$

where *n*_*in*_ is the number of inputs from the previous layer and *n*_*out*_ is the number of outgoing neurons connected to the layer weights. The model was trained using the Adam optimiser^[^ ^43^ ^]^ (β_1_ = 0.9, β_2_ = 0.999, and ϵ = 1 × 10^−7^) with a learning rate of 1 × 10^−3^. A batch size of 16 examples per step was used for training. Following identification of the best architecture (a three layer network comprising 256, 64, and 16 neurons, respectively), tenfold cross validation was performed and the epoch with the lowest mean validation loss across all folds selected as the number of epochs (881) for training the final model (Figure S4A, Supporting Information). The percentage of randomly disabled connections in each Monte Carlo dropout iteration was 50%. The optimum number of Monte Carlo dropout simulations per prediction that gave the lowest MAE overall on the hold‐out test set was 329 (Figure S4B, Supporting Information). Neural networks were coded, trained, and deployed in TensorFlow v. 2.12.1 for Python 3 using a single Nvidia RTX 3080 (8GB) with CUDA 11.8.

### Model Evaluation

Models were evaluated using a hold out test set comprising approximately 10% of the full available dataset for each target attribute. Test set examples were selected at random. Metrics used for evaluation were *R* ^2^:

(5) $$R^{2}=1-\frac{SS_{res}}{SS_{tot}}$$

where *SS*_*res*_ is the residual sum of squared differences and *SS*_*tot*_ is the total sum of squared differences. The mean absolute error (MAE), is also reported, which is more robust to outliers:

(6) $$MAE=\frac{1}{n}\sum_{i=1}^{n}\left|y_{i}-{\hat{y}}_{i}\right|$$

where *n* is the number of observations, *y*_*i*_ is the target value, and ${\hat{y}}_{i}$ is the corresponding predicted value. Regression was used rather than direct classification to avoid information loss. However, when determining model performance relative to a threshold (e.g., PDI < 0.3), a regression model's outputs could be treated like those from a classifier, with average predicted values falling within a specific class boundary treated as predicting that class, and those falling within another boundary as a different class (approach (i)). Alternatively, post hoc classification could also be conducted iteratively (approach (ii)), with each estimators' output assigned to its respective class, and the proportion of estimators in the ensemble choosing a specific class treated as the probability of the target being that class. Regardless of the post hoc classification approach, to evaluate classification performance for PDI thresholds in this study *F*_1_ score was used, which calculates the harmonic mean of precision and recall as:

(7) $$Precision=\frac{TP}{TP+FP}$$

(8) $$Recall=\frac{TP}{TP+FN}$$

where *TP* is the number of true positives, *FP* is the number of false positives, and *FN* is the number of false negatives. Then *F*_1_ can be calculated as:

(9) $$F_{1}=2\frac{Precision\times Recall}{Precision+Recall}$$

For multi‐classification, the macro *F*_1_ is reported which is the mean of the individual *F*_1_ scores obtained for each class versus all others.

### Feature Importance Analysis

For permutation feature importance analysis, the model was trained, then feature columns in the validation set are randomly shuffled in turn before inference. The feature that causes the largest increase in MAE across the validation set when randomised could be considered the most important (Table S2, Supporting Information). Tenfold cross validation for permutation importance analysis was used. For visualization and simplification, the feature importances were min‐max scaled so that the resultant scaled value could be easily read and plotted as the proportional contribution of that feature. To assess the difference in feature importance at N/P ratios above 1 or below 1, two additional permutation analysis experiments were conducted, where N/P ratios above or below this threshold were excluded from the validation set during each round of permutation.

### In Silico Nanocarrier Library Design

Peptide‐dendrimer sequences were randomly generated based on existing sequences that had already been synthesised and tested in the lab. First, the individual sequences of each layer and each core of existing dendrimers were extracted. Any duplicate layer sequences were discarded. This gave a total of 35 unique short sequences from layers and 13 possible cores. All possible combinations of these sequences were then generated, allowing for a core sequence plus any branch sequence in 1 to 3 dendrimer layers. This gave a total of 573,755 possible peptide‐dendrimers (excluding 46 existing peptide‐dendrimers). DLN formulations were then allowed using two different N/P ratios (N/P 0.6 and N/P 8). These N/P ratios showed the highest average transfection performance amongst DLNs formulated at N/P ratios above 1 or below 1. Formulations were then allowed at any of the L/P ratios used in the original dataset (5, 7.5, 10, or 12.5). This resulted in an in silico library of 4,589,672 unique DLN formulations.

### Statistics

Two‐sample, two‐sided, Mann‐Whitney U tests, and calculation of Pearson correlations were performed in Python 3 using SciPy v. 1.10.1.^[^ ^44^ ^]^ For proportions (i.e., the proportion of estimators predicting a specific class), confidence intervals were calculated using:

(10) $$CI=\hat{p}\pm 1.96\sqrt{\frac{\hat{p}\left(1-\hat{p}\right)}{n}}$$

where *CI* is the confidence interval, $\hat{p}$ is the proportion, *n* is the number of samples. As there are many estimators *n* in our ensemble models, we use 1.96, the critical value of the *z* distribution, to approximate 95% confidence. Standard deviations and standard errors reported in this work use the sample standard deviation. Coefficient of variation (CV) was standard deviation divided by the mean. The bandwidth of kernel density estimates (for violin plots) were defined by Scott's rule.^[^ ^45^ ^]^ The bin width for histograms were defined by the Freedman–Diaconis rule.^[^ ^46^ ^]^ In plots where specific P‐Values were not shown ns = not significant, **** = 1e‐4, *** = 1e‐3, ** = 1e‐2, and * = < 0.05. The convention of *P* < 0.05 was used as the threshold for reporting statistically significant differences in this study. Data visualisation was performed using Matplotlib v. 3.7.1^[^ ^47^ ^]^ and Seaborn v. 0.12.2^[^ ^48^ ^]^ for Python 3.

## Conflict of Interest

All authors are employees of Nuntius Therapeutics Limited, of which A.K. and B.N. are co‐founders and directors. This work is associated with patent application PCT/EP2023/074085.

## Author Contributions

T.H.B. performed all computational modelling, data preparation, and the majority of the large‐scale data analyses. L.M., A.L., and M.A. formulated D.L.N. candidates and were responsible for the acquisition of DLS measurements. P.S. and A.L. grew, maintained, and transfected cells, and were responsible for fluorescence and absorbance assays. A.K. and B.N. initiated and directed the project. T.H.B. wrote the manuscript in conjunction with A.K..

## Supporting information

Supporting Information

## Data Availability

The data that support the findings of this study are available from the corresponding author upon reasonable request.

## References

[1] X. Hou , T. Zaks , R. Langer , Y. Dong , Nat. Rev. Mater. 2021, 6, 1078. 34394960 10.1038/s41578-021-00358-0 PMC8353930

[2] Y. Jia , X. Wang , L. Li , F. Li , J. Zhang , X. Liang , Adv. Mater. 2024, 36, 2305300. 10.1002/adma.202305300 37547955

[3] A. Gigante , M. Li , S. Junghänel , C. Hirschhäuser , S. Knauer , C. Schmuck , MedChemComm 2019, 10, 1692. 32180915 10.1039/c9md00275h PMC7053704

[4] F. P. Polack , S. J. Thomas , N. Kitchin , J. Absalon , A. Gurtman , S. Lockhart , J. L. Perez , G. Pérez Marc , E. D. Moreira , C. Zerbini , N. Engl. J. Med. 2020, 383, 2603. 33301246 10.1056/NEJMoa2034577 PMC7745181

[5] L. R. Baden , H. M. El Sahly , B. Essink , K. Kotloff , S. Frey , R. Novak , D. Diemert , S. A. Spector , N. Rouphael , C. B. Creech , N. Engl. J. Med. 2021, 384, 403.33378609

[6] Y. Lee , M. Jeong , J. Park , H. Jung , H. Lee , Exp. Mol. Med. 2023, 55, 2085. 37779140 10.1038/s12276-023-01086-x PMC10618257

[7] D. Loughrey , J. E. Dahlman , Acc. Chem. Res. 2021, 55, 13. 34859663 10.1021/acs.accounts.1c00601

[8] S. A. Dilliard , Q. Cheng , D. J. Siegwart , Proc. Natl. Acad. Sci. 2021, 118, e2109256118. 34933999 10.1073/pnas.2109256118 PMC8719871

[9] Q. Cheng , T. Wei , L. Farbiak , L. T. Johnson , S. A. Dilliard , D. J. Siegwart , Nat. Nanotechnol. 2020, 15, 313. 32251383 10.1038/s41565-020-0669-6 PMC7735425

[10] J. G. Rurik , I. Tombácz , A. Yadegari , P. O. Méndez Fernández , S. V. Shewale , L. Li , T. Kimura , O. Y. Soliman , T. E. Papp , Y. K. Tam , Science 2022, 375, 91. 34990237 10.1126/science.abm0594 PMC9983611

[11] U. Sahin , P. Oehm , E. Derhovanessian , R. A. Jabulowsky , M. Vormehr , M. Gold , D. Maurus , D. Schwarck‐Kokarakis , A. N. Kuhn , T. Omokoko , Nature 2020, 585, 107. 32728218 10.1038/s41586-020-2537-9

[12] L. M. Kranz , M. Diken , H. Haas , S. Kreiter , C. Loquai , K. C. Reuter , M. Meng , D. Fritz , F. Vascotto , H. Hefesha , Nature 2016, 534, 396. 27281205 10.1038/nature18300

[13] C. Krienke , L. Kolb , E. Diken , M. Streuber , S. Kirchhoff , T. Bukur , Ö. Akilli‐Öztärk , L. M. Kranz , H. Berger , J. Petschenka , Science 2021, 371, 145. 33414215 10.1126/science.aay3638

[14] K. Zhou , L. H. Nguyen , J. B. Miller , Y. Yan , P. Kos , H. Xiong , L. Li , J. Hao , J. T. Minnig , H. Zhu , D. J. Siegwart , Proc. Natl. Acad. Sci. 2016, 113, 520. 26729861 10.1073/pnas.1520756113 PMC4725465

[15] A. Kwok , G. A. Eggimann , J.‐L. Reymond , T. Darbre , F. Hollfelder , ACS Nano 2013, 7, 4668. 23682947 10.1021/nn400343z PMC3715887

[16] Q. Cheng , T. Wei , Y. Jia , L. Farbiak , K. Zhou , S. Zhang , Y. Wei , H. Zhu , D. J. Siegwart , Adv. Mater. 2018, 30, 1805308. 10.1002/adma.201805308 30368954

[17] L. Farbiak , Q. Cheng , T. Wei , E. Álvarez‐Benedicto , L. T. Johnson , S. Lee , D. J. Siegwart , Adv. Mater. 2021, 33, 2006619. 10.1002/adma.202006619 PMC10041668 34137093

[18] A. Kwok , G. A. Eggimann , M. Heitz , J. Reymond , F. Hollfelder , T. Darbre , ChemBioChem 2016, 17, 2223. 27862758 10.1002/cbic.201600485

[19] M. Heitz , A. Kwok , G. A. Eggimann , F. Hollfelder , T. Darbre , J.‐L. Reymond , Chimia 2017, 71, 220. 28446340 10.2533/chimia.2017.220

[20] A. Kwok , S. L. Hart , Nanomedicine Nanotechnol. Biol. Med. 2011, 7, 210.

[21] A. S. Chauhan , Molecules 2018, 23, 938.29670005

[22] V. L. Herrera , A. H. Colby , N. Ruiz‐Opazo , D. G. Coleman , M. W. Grinstaff , Nanomed. 2018, 13, 2083. 10.2217/nnm-2018-0122 PMC6219437 30204054

[23] A. Sarode , Y. Fan , A. E. Byrnes , M. Hammel , G. L. Hura , Y. Fu , P. Kou , C. Hu , F. I. Hinz , J. Roberts , Nanoscale Adv. 2022, 4, 2107. 36133441 10.1039/d1na00712b PMC9417559

[24] L. Cui , S. Pereira , S. Sonzini , S. van Pelt , S. M. Romanelli , L. Liang , D. Ulkoski , V. R. Krishnamurthy , E. Brannigan , C. Brankin , Nanoscale 2022, 14, 1480. 35024714 10.1039/d1nr06858j

[25] T. P. Hopp , K. R. Woods , Proc. Natl. Acad. Sci. 1981, 78, 3824. 6167991 10.1073/pnas.78.6.3824 PMC319665

[26] M. Danaei , M. Dehghankhold , S. Ataei , F. Hasanzadeh Davarani , R. Javanmard , A. Dokhani , S. Khorasani , M. Mozafari , Pharmaceutics 2018, 10, 57. 29783687 10.3390/pharmaceutics10020057 PMC6027495

[27] P. Blasi , S. Giovagnoli , A. Schoubben , M. Ricci , C. Rossi , Adv. Drug Deliv. Rev. 2007, 59, 454. 17570559 10.1016/j.addr.2007.04.011

[28] L. A. S. Bahari , H. Hamishehkar , Adv. Pharm. Bull. 2016, 6, 143. 27478775 10.15171/apb.2016.021 PMC4961971

[29] N. Bertrand , J.‐C. Leroux , J. Controlled Release 2012, 161, 152. 10.1016/j.jconrel.2011.09.098 22001607

[30] M. I. Townsley , J. C. Parker , G. L. Longenecker , M. L. Perry , R. M. Pitt , A. E. Taylor , Am. J. Physiol.‐Heart Circ. Physiol. 1988, 255, H1075. 10.1152/ajpheart.1988.255.5.H1075 3189571

[31] C. H. J. Choi , J. E. Zuckerman , P. Webster , M. E. Davis , Proc. Natl. Acad. Sci. 2011, 108, 6656. 21464325 10.1073/pnas.1103573108 PMC3080986

[32] J. C. Kraft , J. P. Freeling , Z. Wang , R. J. Ho , J. Pharm. Sci. 2014, 103, 29. 24338748 10.1002/jps.23773 PMC4074410

[33] H. Sarin , J. Angiogenesis Res. 2010, 2, 1. 10.1186/2040-2384-2-14 PMC2928191 20701757

[34] S. Ogawa , Z. Ota , K. Shikata , K. Hironaka , Y. Hayashi , K. Ota , M. Kushiro , N. Miyatake , N. Kishimoto , H. Makino , Am. J. Nephrol. 1999, 19, 686. 10592365 10.1159/000013543

[35] L. Breiman , Mach. Learn. 2001, 45, 5.

[36] Y. Gal , Z. Ghahramani , PMLR 2016, 48, 1050.

[37] P. Kos , U. Lächelt , A. Herrmann , F. M. Mickler , M. Döblinger , D. He , A. K. Levačić , S. Morys , C. Bräuchle , E. Wagner , Nanoscale 2015, 7, 5350. 25721131 10.1039/c4nr06556e

[38] S. Honary , F. Zahir , Trop. J. Pharm. Res. 2013, 12, 255.

[39] N. J. Anthis , G. M. Clore , Protein Sci. 2013, 22, 851. 23526461 10.1002/pro.2253 PMC3690723

[40] S. C. Gill , P. H. Von Hippel , Anal. Biochem. 1989, 182, 319. 2610349 10.1016/0003-2697(89)90602-7

[41] F. Pedregosa , G. Varoquaux , A. Gramfort , V. Michel , B. Thirion , O. Grisel , M. Blondel , P. Prettenhofer , R. Weiss , V. Dubourg , J. Mach. Learn. Res. 2011, 12, 2825.

[42] X. Glorot , Y. Bengio , PMLR 2010, 9, 249.

[43] D. P. Kingma , J. Ba , ArXiv Preprint 2014, https://arxiv.org/abs/1412.6980.

[44] P. Virtanen , R. Gommers , T. E. Oliphant , M. Haberland , T. Reddy , D. Cournapeau , E. Burovski , P. Peterson , W. Weckesser , J. Bright , S. J. van der Walt , M. Brett , J. Wilson , K. J. Millman , N. Mayorov , A. R. J. Nelson , E. Jones , R. Kern , E. Larson , C. J. Carey , İ. Polat , Y. Feng , E. W. Moore , J. VanderPlas , D. Laxalde , J. Perktold , R. Cimrman , I. Henriksen , E. A. Quintero , C. R. Harris , et al., Nat. Methods 2020, 17, 261. 32015543 10.1038/s41592-019-0686-2 PMC7056644

[45] D. W. Scott , Wiley Interdiscip. Rev. Comput. Stat. 2009, 1, 303. 10.1002/wics.45 PMC2898213 20625470

[46] D. Freedman , P. Diaconis , Z. Fär Wahrscheinlichkeitstheorie Verwandte Geb. 1981, 57, 453.

[47] J. D. Hunter , Comput. Sci. Eng. 2007, 9, 90.

[48] M. L. Waskom , J. Open Source Softw. 2021, 6, 3021.

