# Supplementary material for: In Silico Screening Accelerates Nanocarrier Design for Efficient mRNA Delivery
Source: Adv Sci (Weinh). 2024 Jun 5;11(30):2401935. doi: 10.1002/advs.202401935 (PMC11321627; doi:10.1002/advs.202401935)
Supplement: Supplementary file 1 — Supporting Information [file ADVS-11-2401935-s001.pdf]

ADVANCED SCIENCE
Open Access

Supporting Information

for *Adv. Sci.*, DOI 10.1002/advs.202401935

In Silico Screening Accelerates Nanocarrier Design for Efficient mRNA Delivery

Tristan Henser-Brownhill, Liam Martin, Parisa Samangouei, Aaqib Ladak, Marina Apostolidou, Benita Nagel and Albert Kwok**

SUPPLEMENTARY FIGURES & TABLES

In silico Screening Accelerates Nanocarrier Design for Efficient mRNA Delivery

[1,*]Tristan Henser-Brownhill, [1]Liam Martin, [1]Parisa Samangouei, [1]Aaqib Ladak, [1]Marina Apostolidou, [1]Benita Nagel, and [1,*,†]Albert Kwok

[1]Nuntius Therapeutics Limited, London, W10 5JJ
[*]Correspondence to: albert@nuntiustx.com and tristan@nuntiustx.com
[†]Lead contact

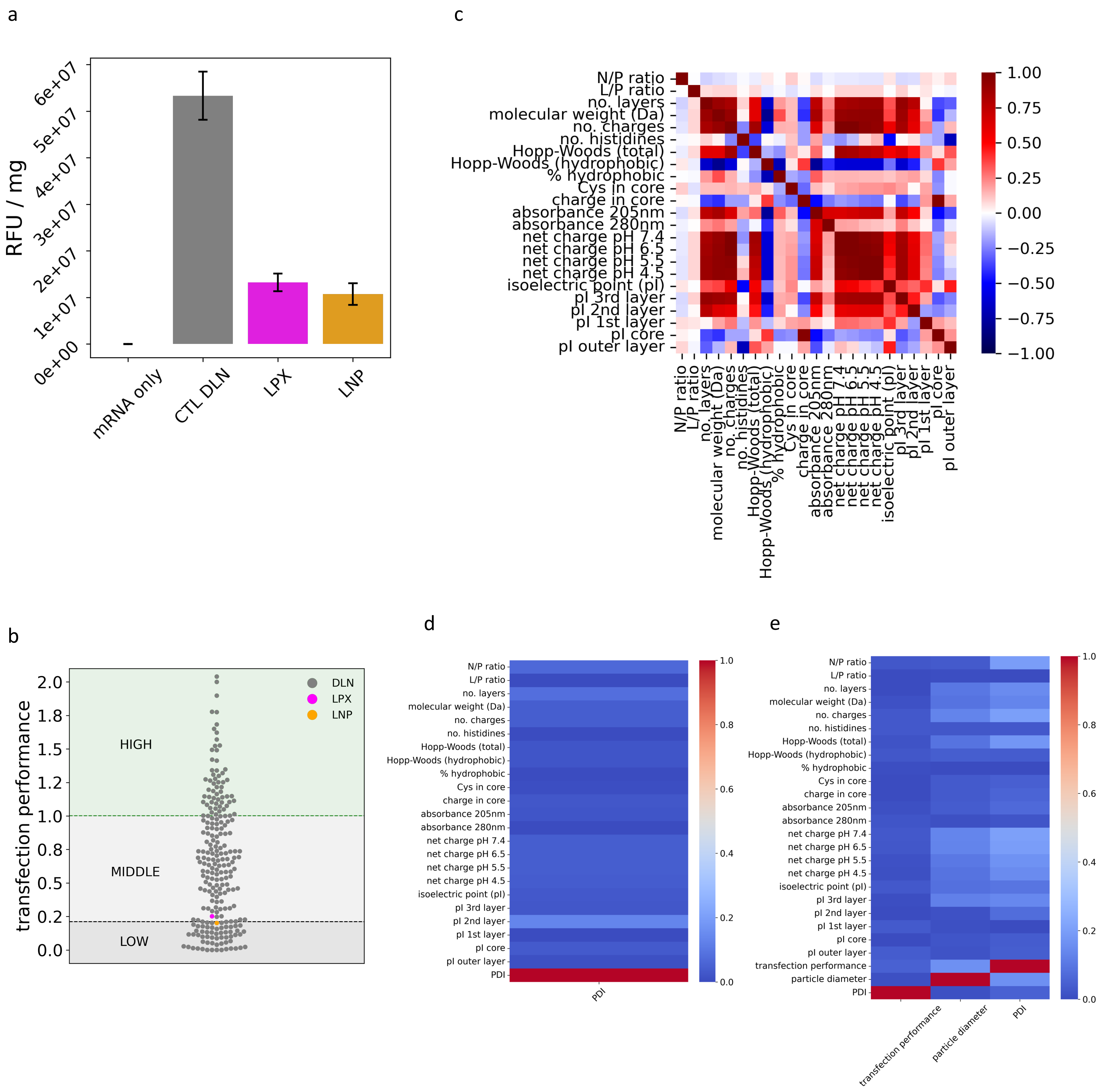

a
RFU / mg
0e+00
1e+07
2e+07
3e+07
4e+07
5e+07
6e+07
mRNA only
CTL DLN
LPX
LNP
b
transfection performance
HIGH
MIDDLE
LOW
DLN
LPX
LNP
c
N/P ratio
L/P ratio
no. layers
molecular weight (Da)
no. charges
no. histidines
Hopp-Woods (total)
Hopp-Woods (hydrophobic)
% hydrophobic
Cys in core
charge in core
absorbance 205nm
absorbance 280nm
net charge pH 7.4
net charge pH 6.5
net charge pH 5.5
net charge pH 4.5
isoelectric point (pI)
pI 3rd layer
pI 2nd layer
pI 1st layer
pI core
pI outer layer
d
PDI
e
transfection performance
particle diameter
PDI

Figure S1. Lipidic nanocarrier comparison and feature correlations.

A. Comparison between an optimised DLN positive control (from prior laboratory screening) and two state-of-the-art LPX[11,12] and LNP[9] formulations (see methods). Error bars are standard deviation across 6 replicates.

B. Swarm plot showing the normalised transfection performance of all DLNs in our dataset with PDI $\leq$ 0.3 compared to two state-of-the-art LPX[11,12] and LNP[9] formulations. Transfection performance classes are also visualised as colour bands.

C. Pearson correlation matrix of all 23 features used in this study visualised as a heatmap; 1 (red) would represent a perfect positive correlation and -1 (blue) a perfect negative correlation. Values close to 0 (white) indicate no correlation at all.

D-E. Heatmaps showing the R^2 for each of our 23 features versus our targets of interest; **C.** shows all data, where **D.** only shows data for DLNs with PDI $\leq$ 0.3. Note that in this case the R^2 is simply the square of the Pearson correlation coefficient and not the same as the coefficient of determination used for model evaluation (see methods).

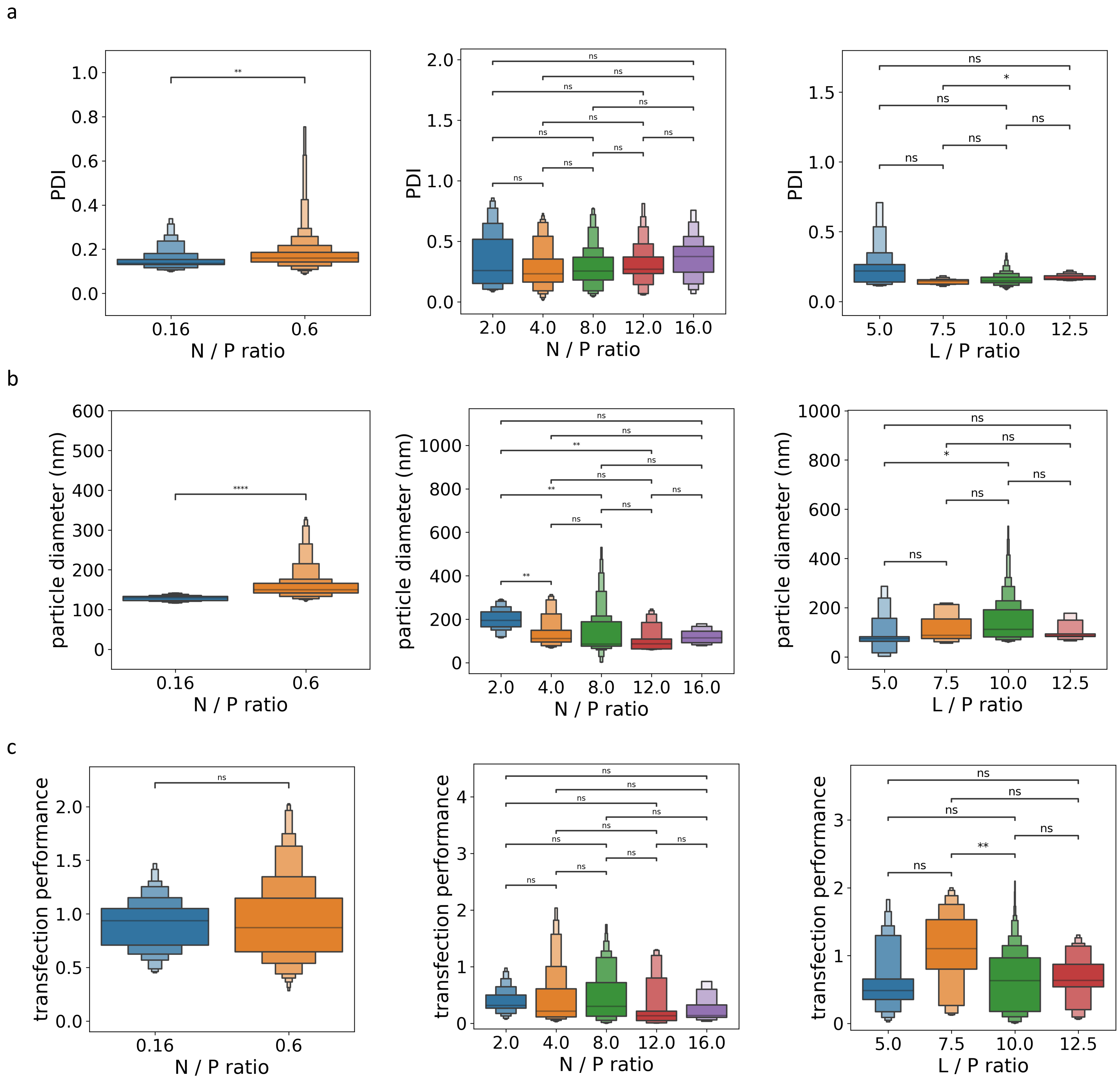

a
PDI
N / P ratio
L / P ratio
b
particle diameter (nm)
N / P ratio
L / P ratio
c
transfection performance
N / P ratio
L / P ratio

Figure S2. DLN component ratios compared to our targets of interest.

A. Boxenplots showing N/P or L/P ratios versus PDI for all DLNs in the dataset.

B-C. Boxenplots showing N/P or L/P ratios versus particle diameter (nm) and transfection performance, respectively, for all DLNs in the dataset with PDI $\leq$ 0.3. The line in the centre of each box represents the median, with each segment representing a quartile. We used 'full' k_depth (log2(n) + 1 levels). Statistical comparison between target value distributions are non-parametric Mann-Whitney U tests.

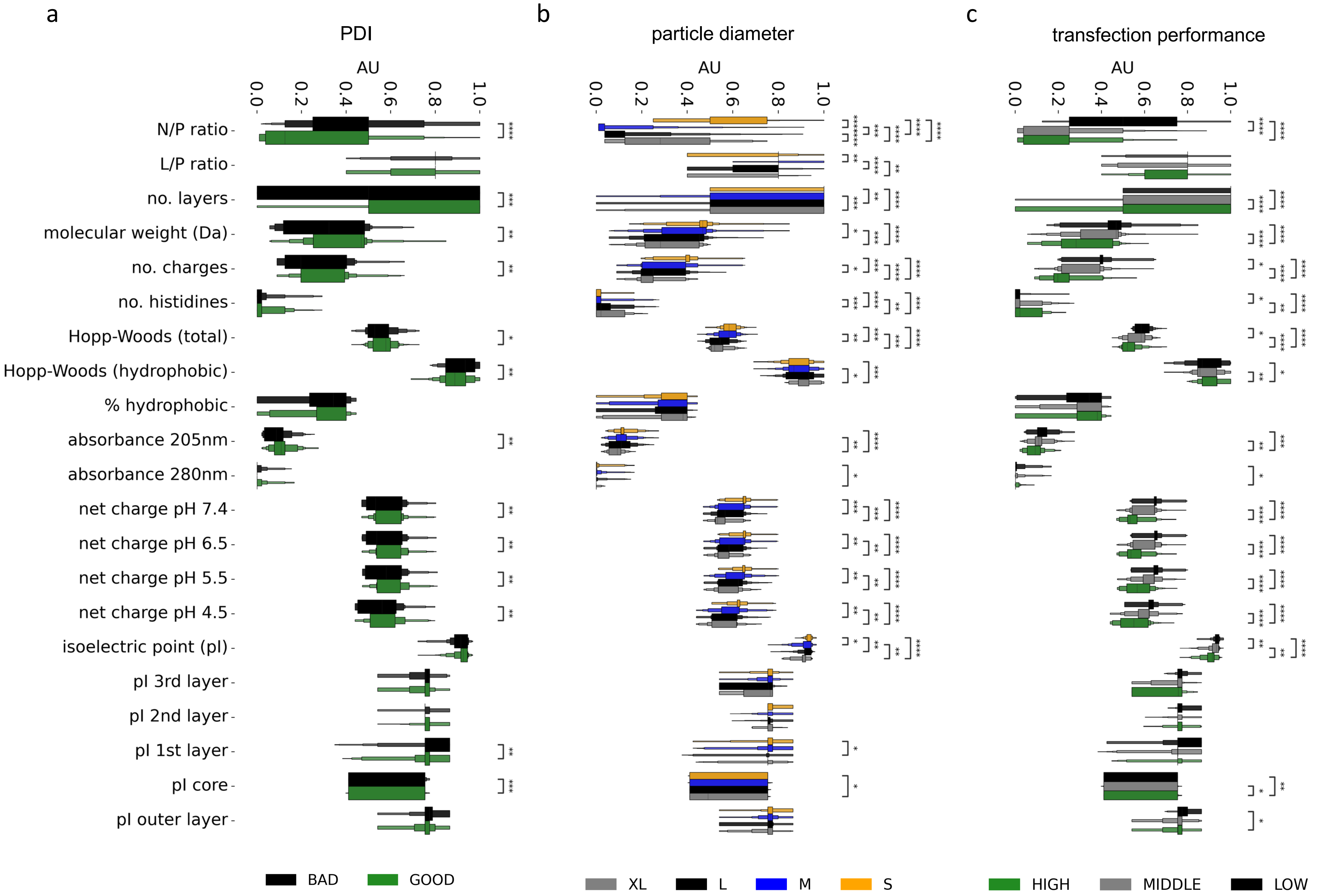

a
PDI
AU
0.0
0.2
0.4
0.6
0.8
1.0
N/P ratio
L/P ratio
no. layers
molecular weight (Da)
no. charges
no. histidines
Hopp-Woods (total)
Hopp-Woods (hydrophobic)
% hydrophobic
absorbance 205nm
absorbance 280nm
net charge pH 7.4
net charge pH 6.5
net charge pH 5.5
net charge pH 4.5
isoelectric point (pI)
pI 3rd layer
pI 2nd layer
pI 1st layer
pI core
pI outer layer
BAD
GOOD
b
particle diameter
AU
XL
L
M
S
c
transfection performance
AU
HIGH
MIDDLE
LOW

Figure S3. Comparison of feature distributions with target classes.

A. 21 continuous features visualised as boxplots showing differences and similarities between each of the PDI categories. Data for all DLNs is shown.

B-C. 21 continuous features visualised as boxplots showing differences and similarities between each of the **B.** particle diameter or **C.** transfection performance categories. Data for all DLNs with PDI $\leq$ 0.3 is shown. Statistical comparisons between feature distributions are non-parametric Mann-Whitney U tests.

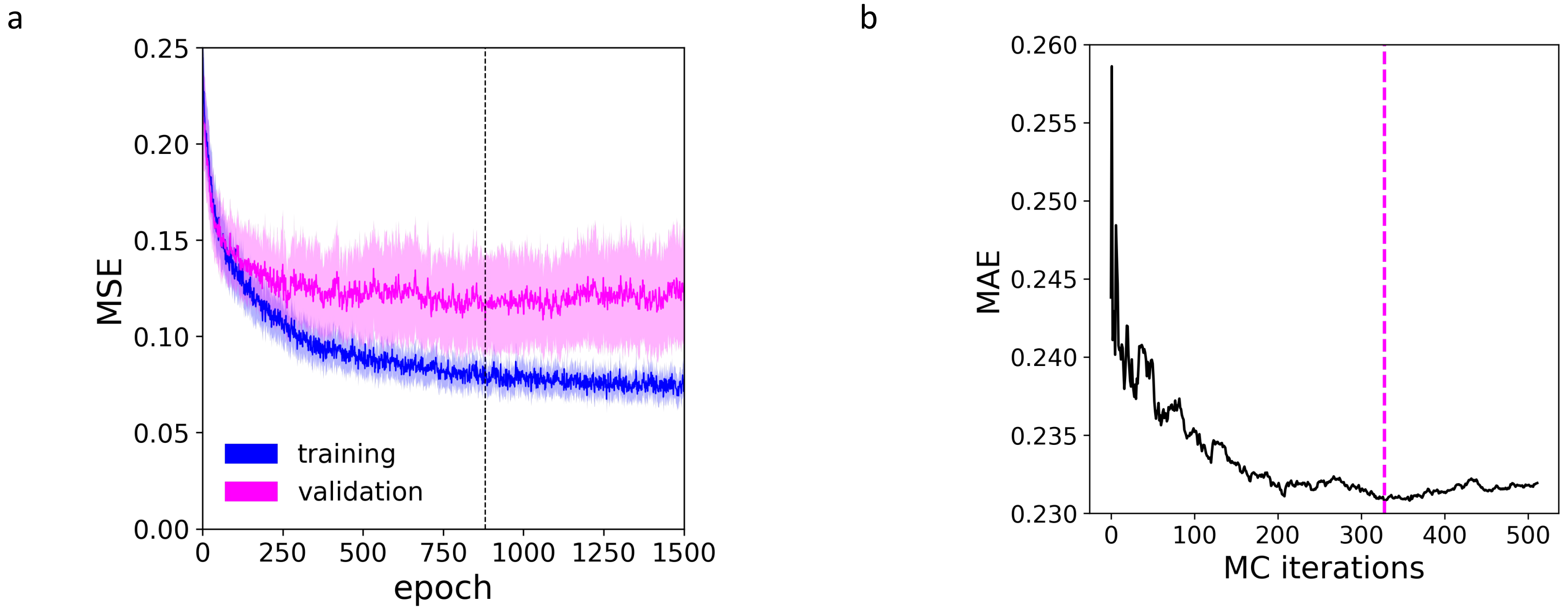
a
MSE
epoch
training
validation
0.25
0.20
0.15
0.10
0.05
0.00
0
250
500
750
1000
1250
1500
b
MAE
MC iterations
0.260
0.255
0.250
0.245
0.240
0.235
0.230
0
100
200
300
400
500

Figure S4. Neural network training and validation.

A. Training and validation curves for our transfection performance neural network model. Results are from 10-fold cross validation on the training set. Error margins are standard error. The dashed line indicated the lowest mean squared error (MSE) validation loss and the epoch at which we invoked early stopping during final training of the model to avoid overfitting.

B. Plot showing the number of Monte Carlo (MC) dropout iterations used versus the MAE obtained on the independent test set, demonstrating that this approach continues to improve predictive performance until around 329 iterations (dashed line).

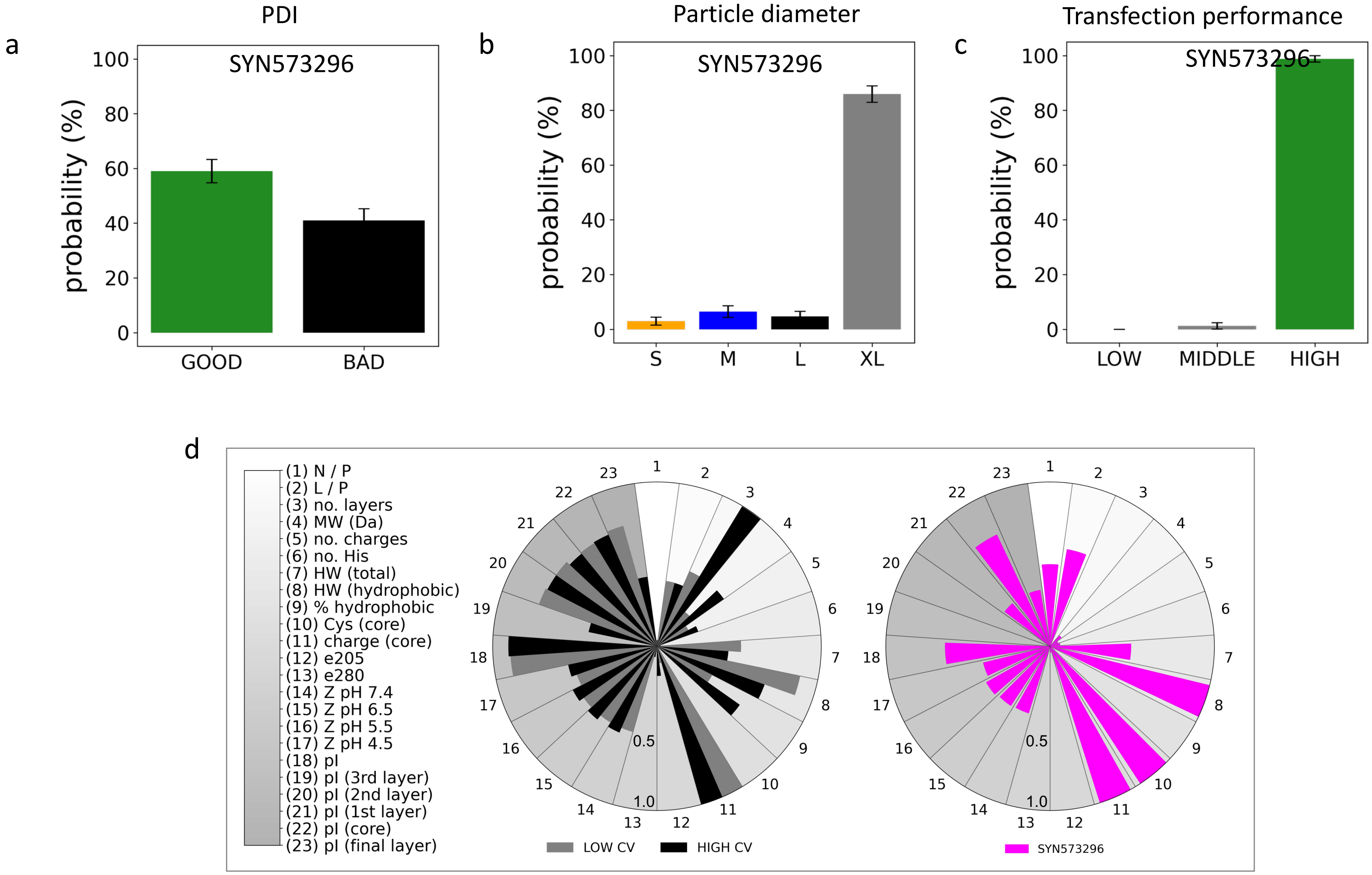
a
PDI
SYN573296
probability (%)
GOOD
BAD
b
Particle diameter
SYN573296
probability (%)
S
M
L
XL
c
Transfection performance
SYN573296
probability (%)
LOW
MIDDLE
HIGH
d
(1) N / P
(2) L / P
(3) no. layers
(4) MW (Da)
(5) no. charges
(6) no. His
(7) HW (total)
(8) HW (hydrophobic)
(9) % hydrophobic
(10) Cys (core)
(11) charge (core)
(12) e205
(13) e280
(14) Z pH 7.4
(15) Z pH 6.5
(16) Z pH 5.5
(17) Z pH 4.5
(18) pI
(19) pI (3rd layer)
(20) pI (2nd layer)
(21) pI (1st layer)
(22) pI (core)
(23) pI (final layer)
LOW CV
HIGH CV
SYN573296

Figure S5. ***In silico*** **candidate predictions.**

A-C. The proportion of individual model estimators where the predicted value fell within the two predefined PDI classes, four particle diameter classes, or three transfection performance classes for the three selected *in silico* predicted candidates. Plots represent the predicted probability of a particular class. Error bars represent 95% proportional confidence intervals.

D. Polar bar plots showing mean min-max scaled feature values for: (left) the top 1000 DLN candidates with the highest predicted transfection performance grouped by low or high CV; (right) candidate SYN573296 selected for real-world synthesis and evaluation.

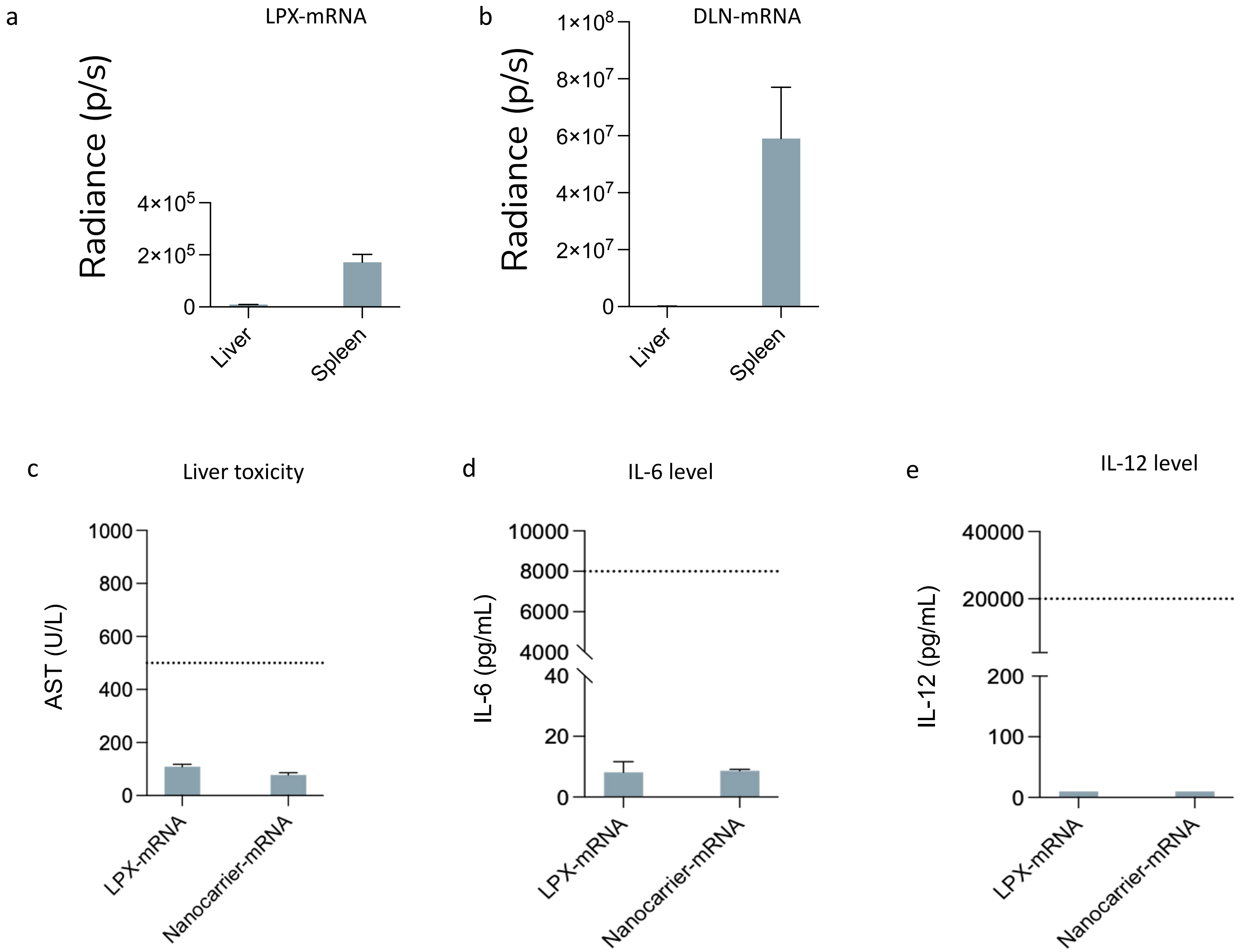

a
LPX-mRNA
Radiance (p/s)
4×10^5
2×10^5
0
Liver
Spleen
b
DLN-mRNA
Radiance (p/s)
1×10^8
8×10^7
6×10^7
4×10^7
2×10^7
0
Liver
Spleen
c
Liver toxicity
AST (U/L)
1000
800
600
400
200
0
LPX-mRNA
Nanocarrier-mRNA
d
IL-6 level
IL-6 (pg/mL)
10000
8000
6000
4000
40
20
0
LPX-mRNA
Nanocarrier-mRNA
e
IL-12 level
IL-12 (pg/mL)
40000
20000
200
100
0
LPX-mRNA
Nanocarrier-mRNA

Figure S6. ***In vivo*** **delivery and toxicity evaluation.**

A-B. Payload reporter (fLuc) expression in liver and spleen tissue when delivered via **A.** state-of-the-art myeloid cell targeting lipoplexes (LPX)[11,12] or **B.** our DLN nanocarriers. Delivered intravenously to wild type female mice.

C-E. Liver toxicity and immunogenicity (cytokine response) measured after intravenous delivery of myeloid cell targeting LPX[11,12] (left) or our DLN nanocarriers (right). Aspartate aminotransferase (AST) correlates with liver toxicity. IL-6 and IL-12 levels indicate immune activation. Error bars show standard errors.

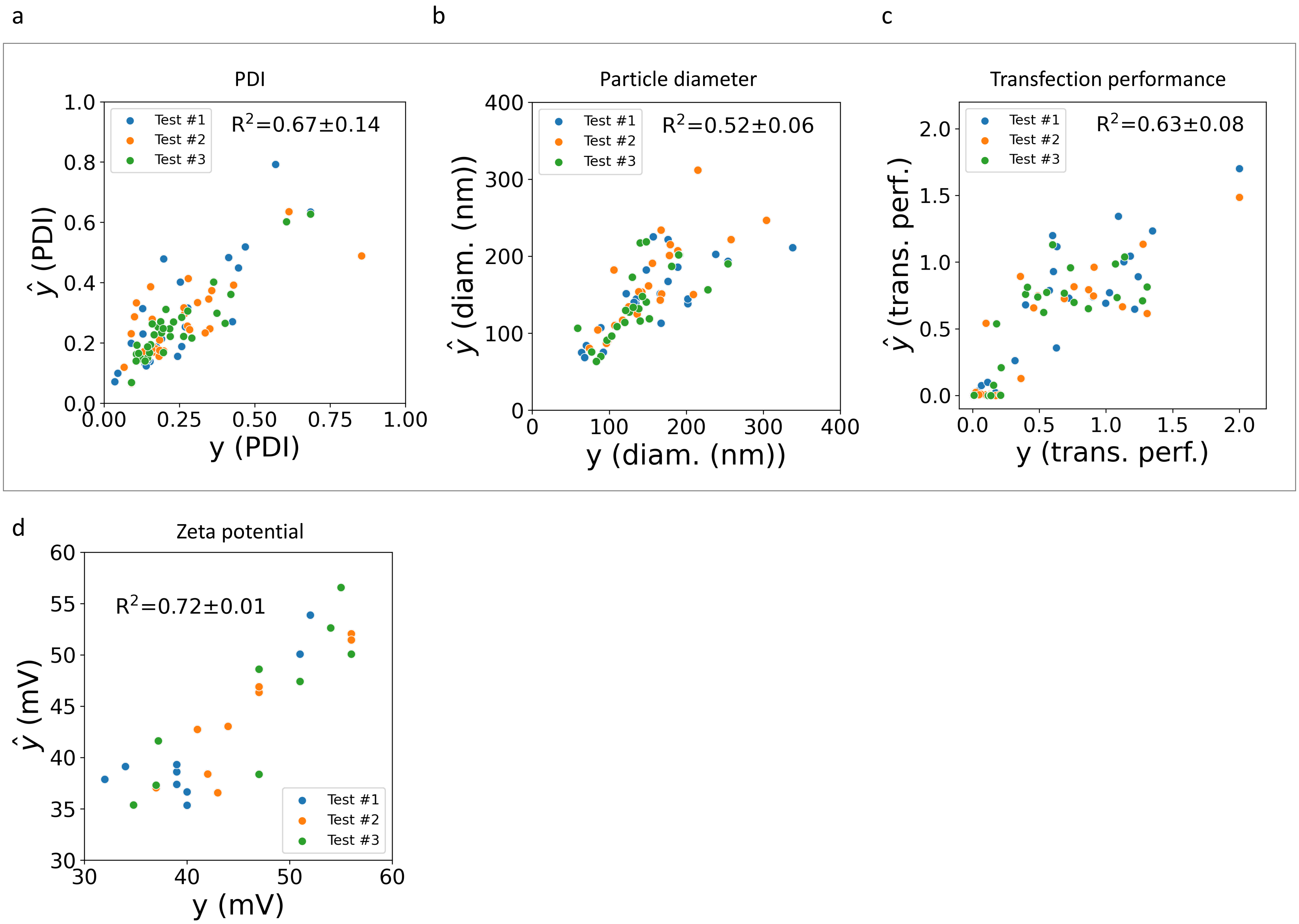
a
PDI
R^2=0.67±0.14
Test #1
Test #2
Test #3
ŷ (PDI)
y (PDI)
0.0
0.2
0.4
0.6
0.8
1.0
0.00
0.25
0.50
0.75
1.00
b
Particle diameter
R^2=0.52±0.06
Test #1
Test #2
Test #3
ŷ (diam. (nm))
y (diam. (nm))
0
100
200
300
400
c
Transfection performance
R^2=0.63±0.08
Test #1
Test #2
Test #3
ŷ (trans. perf.)
y (trans. perf.)
0.0
0.5
1.0
1.5
2.0
d
Zeta potential
R^2=0.72±0.01
Test #1
Test #2
Test #3
ŷ (mV)
y (mV)
30
35
40
45
50
55
60

Figure S7. Additional model validation and zeta potential prediction modelling results.

A-C. Additional evaluation of performance of our three ML models. Each colour shows a model trained and then tested on a different random train-test (90% vs. 10%) split.

D. Evaluation of a random forest (RF) model trained to predict zeta potential (mV). Results are from three random train-test (90% vs 10%) splits. The zeta potential dataset contained 89 example measurements.

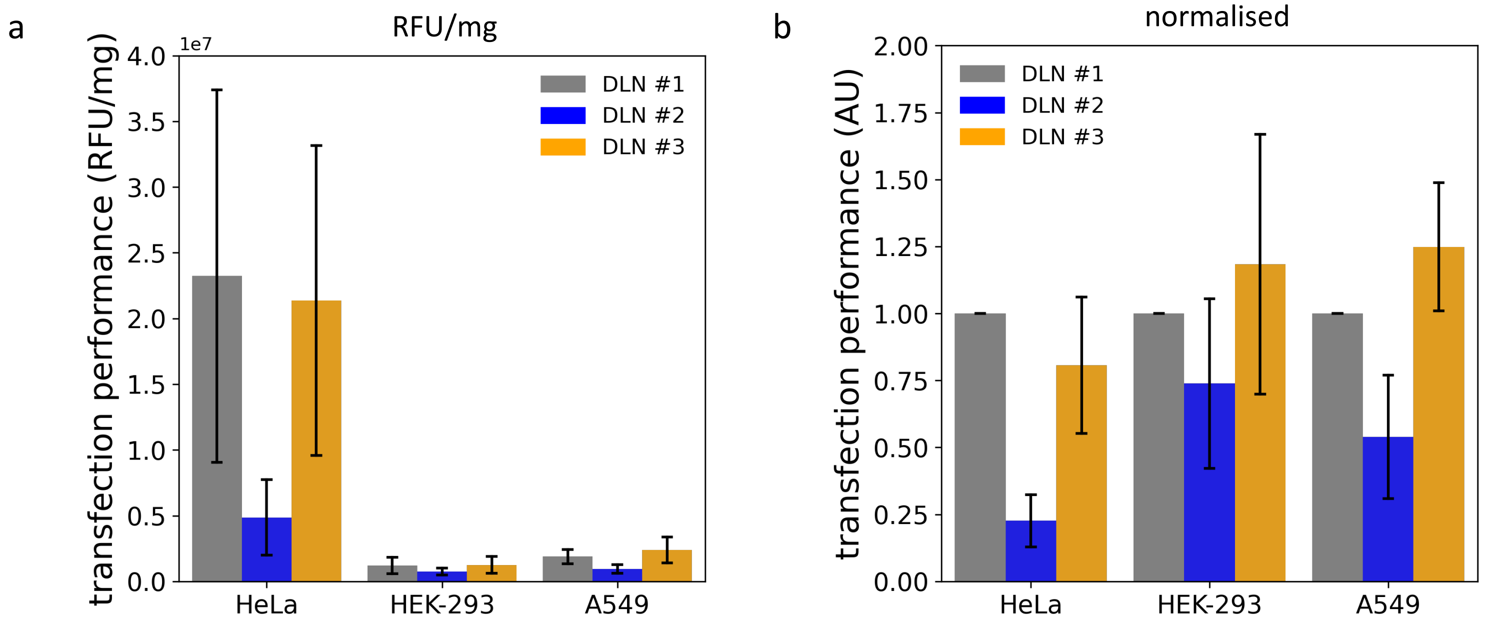
a
RFU/mg
1e7
transfection performance (RFU/mg)
4.0
3.5
3.0
2.5
2.0
1.5
1.0
0.5
0.0
DLN #1
DLN #2
DLN #3
HeLa
HEK-293
A549
b
normalised
transfection performance (AU)
2.00
1.75
1.50
1.25
1.00
0.75
0.50
0.25
0.00
DLN #1
DLN #2
DLN #3
HeLa
HEK-293
A549

Figure S8. Multi-cell line evaluation.

Evaluation of our three control DLNs (CTL1, 2 and 3) across three separate human cell lines. **A.** shows RFU/mg protein, demonstrating that A549 and HEK-293 cells are generally harder to transfect than HeLa. **B.** shows DLNs normalised to the primary positive control formulation (CTL1); this is less variable between different plates, cell lines, and experiments permitting better comparison between these. Error bars show standard deviation.

Supplementary Table S1

**Feature name**	**Description**	**Min | Max**
N/P ratio	The ratio of positively charged amine nitrogen (N) in the peptide-dendrimer to phosphate in the nucleic acid payload.	0 | 16
L/P ratio	The w:w ratio of lipid components to the nucleic acid payload.	0 | 12.5
No. layers	The number of generations / layers comprising the branched peptide-dendrimer.	1 | 3
Molecular weight (Da)	Molecular weight of the peptide-dendrimer component	296 | 10209
No. charges	The number of positively charged side chains in the dendrimer's peptide sequence.	0 | 56
No. Histidines	The total number of histidine residues in the dendrimer's peptide sequence.	0 | 48
Hopp-Woods (total)	The sum of the Hopp-Woods hydrophilicity scores from each residue in the peptide-dendrimer – Hopp and Woods (1981).	-142.2 | 165
Hopp-Woods (hydrophobic)	The sum of the Hopp-Woods hydrophilicity scores from each hydrophobic residue in the peptide-dendrimer.	-163.2 | 0
% hydrophobic	The percentage of residues comprising the dendrimer's peptide sequence that are classed as hydrophobic.	0, 1
Cystine (core)	Whether a cysteine residue resides in the dendrimer's core sequence.	N/A
Charge (core)	Whether a positively charged side chain is present in the dendrimer's core sequence.	N/A
A205 / e205	Computational estimation of absorbance of the dendrimer component at 205 nm, based on Anthis and Clore (2013).	0 | 1129320
A280 / e280	Computational estimation of absorbance of the dendrimer component at 280 nm, based on Gill and von Hippel (1989).	0 | 264000
Net charge (Z) at pH 7.4	The net charge Z of the dendrimer component at pH 7.4 estimated from the amino acid composition using: $$Z = \sum_i N_i \frac{10^{pKa_i}}{10^{pH} + 10^{pKa_i}} - \sum_j N_j \frac{10^{pH}}{10^{pH} + 10^{pKa_j}}$$ Where N_i are the total quantities of Arginine, Lysine, and Histidine, and pKa_i the pKa of their side chains and N-termini; and N_j are the total quantities of Aspartic Acid, Glutamic Acid, Cysteine, Tyrosine, and pKa_j the pKa of their side chains and C-termini.	-40.04 | 55.83

Supplementary Table S1 (cont.)

Net charge (Z) at pH 6.5	As above, but for pH 6.5.	-39.93 | 55.98
Net charge (Z) at pH 5.5	As above, but for pH 5.5.	-39.23 | 56
Net charge (Z) at pH 4.5	As above, but for pH 4.5.	-33.3 | 56
pI	The isoelectric point of the dendrimer component, which is the pH at which the net charge is closest to 0. Estimated computationally by incrementing the pH by 0.01 between 0 and 14.	0, 14
pI g3	As above, but only for amino acids in the third generation of the dendrimer. If no third generation is present, the model receives the value -1 after feature scaling, indicating a missing value.	0, 14
pI g2	As above, but only for amino acids in the second generation of the dendrimer. If no second generation is present, the model receives the value -1 after feature scaling, indicating a missing value.	0, 14
pI g1	As above, but only for amino acids in the first generation of the dendrimer.	0, 14
pI core	As above, but only for amino acids comprising the dendrimer core sequence.	0, 14
pI fg	As above, but only for amino acids in the final, outermost, generation of the dendrimer.	0, 14

Table S1. Features used in the study, their computation, and details.

	PDI						Particle diameter						Transfection performance					
Feature	increase_MAE	std	prop_inc_NP	prop_excl_NP	prop_NP_<_1	prop_NP_>_1	increase_MAE	std	prop_inc_NP	prop_excl_NP	prop_NP_<_1	prop_NP_>_1	increase_MAE	std	prop_inc_NP	prop_excl_NP	prop_NP_<_1	prop_NP_>_1
N/P ratio	0.04322	0.01242	0.64990	NA	NA	NA	22.91814	9.51945	0.71887	NA	NA	NA	0.16466	0.04484	0.51513	NA	NA	NA
Cystine (core)	-0.00037	0.00053	0.00000	0.00000	0.00000	0.00000	-0.00312	0.97499	0.00000	0.00000	0.00000	0.00000	0.01112	0.01954	0.03480	0.07176	0.04215	0.10511
Charge (core)	-0.00008	0.00043	0.00000	0.00000	0.00000	0.00000	-0.14402	0.16817	0.00000	0.00000	0.00000	0.00000	-0.00026	0.01801	0.00000	0.00000	0.01714	0.00000
A205 / e205	0.00018	0.00155	0.00275	0.00785	0.00000	0.00000	0.11134	0.60467	0.00349	0.01242	0.00000	0.00000	0.00173	0.00551	0.00543	0.01119	0.00000	0.02123
A280 / e280	-0.00040	0.00103	0.00000	0.00000	0.00931	0.00000	0.08087	2.14000	0.00254	0.00902	0.00000	0.00000	0.00311	0.01287	0.00973	0.02008	0.11540	0.03939
Hopp-Woods (total)	0.00122	0.00334	0.01830	0.05228	0.00000	0.08994	3.99284	3.69743	0.12524	0.44551	0.00000	0.68311	-0.00411	0.01375	0.00000	0.00000	0.00000	0.00823
Hopp-Woods (hydrophobic)	-0.00004	0.00167	0.00000	0.00000	0.00000	0.02646	-0.19361	0.48739	0.00000	0.00000	0.00000	0.02852	0.00369	0.00692	0.01154	0.02380	0.04457	0.00000
L/P ratio	0.00327	0.00469	0.04923	0.14061	0.02491	0.14986	2.01110	4.17442	0.06308	0.22439	0.96671	0.00000	0.00093	0.00724	0.00290	0.00598	0.13554	0.00000
Molecular weight (Da)	0.00025	0.00376	0.00378	0.01080	0.01001	0.08816	-0.06043	0.57157	0.00000	0.00000	0.00000	0.03533	-0.00029	0.00325	0.00000	0.00000	0.00000	0.02346
No. charges	0.00388	0.00343	0.05833	0.16660	0.15279	0.21403	1.13304	1.26590	0.03554	0.12642	0.00000	0.01165	0.02059	0.01944	0.06443	0.13287	0.09205	0.13898
No. layers	-0.00005	0.00047	0.00000	0.00000	0.00917	0.00140	-0.01916	0.05728	0.00000	0.00000	0.00000	0.00000	0.00314	0.00786	0.00982	0.02025	0.11299	0.02410
Net charge (Z) at pH 4.5	0.00057	0.00149	0.00860	0.02457	0.00000	0.00000	0.08033	0.18753	0.00252	0.00896	0.00000	0.00967	0.00180	0.00440	0.00562	0.01159	0.01605	0.00748
Net charge (Z) at pH 5.5	0.00018	0.00168	0.00278	0.00794	0.00000	0.00000	-0.07758	0.42210	0.00000	0.00000	0.00000	0.01193	0.00044	0.00251	0.00138	0.00284	0.01469	0.00000
Net charge (Z) at pH 6.5	0.00048	0.00140	0.00720	0.02056	0.00000	0.00277	0.16811	0.84321	0.00527	0.01876	0.00000	0.06303	0.00093	0.00322	0.00291	0.00600	0.00144	0.00701
Net charge (Z) at pH 7.4	0.00623	0.00709	0.09362	0.26742	0.40566	0.31665	1.13261	1.60996	0.03553	0.12637	0.00000	0.13211	0.00469	0.00859	0.01467	0.03025	0.01341	0.00132
% hydrophobic	0.00028	0.00191	0.00421	0.01203	0.13245	0.00000	-0.78057	0.88874	0.00000	0.00000	0.00000	0.01359	0.00688	0.01889	0.02153	0.04440	0.12797	0.18194
pI	0.00209	0.00248	0.03142	0.08975	0.00000	0.00329	-0.29206	0.66200	0.00000	0.00000	0.00000	0.00000	0.00384	0.00835	0.01200	0.02475	0.06389	0.00205
pI core	-0.00065	0.00127	0.00000	0.00000	0.00000	0.00000	-0.60062	1.46118	0.00000	0.00000	0.00000	0.00000	-0.00046	0.00763	0.00000	0.00000	0.01870	0.01386
pI_fg	-0.00079	0.00130	0.00000	0.00000	0.18611	0.00000	-0.14160	0.28169	0.00000	0.00000	0.00000	0.00000	0.00207	0.01164	0.00648	0.01337	0.03456	0.00392
pI g1	0.00281	0.00316	0.04219	0.12051	0.00000	0.06315	0.22046	0.57527	0.00692	0.02460	0.03329	0.00000	0.00080	0.00842	0.00251	0.00518	0.02218	0.01192
pI g2	0.00173	0.00211	0.02601	0.07431	0.06959	0.03609	-0.10925	0.32370	0.00000	0.00000	0.00000	0.00000	-0.00018	0.02784	0.00000	0.00000	0.04372	0.02884
pI g3	-0.00031	0.00055	0.00000	0.00000	0.00000	0.00000	-0.13300	0.32411	0.00000	0.00000	0.00000	0.00000	0.04856	0.03189	0.15191	0.31330	0.00000	0.33370
No. Histidines	0.00011	0.00127	0.00168	0.00479	0.00000	0.00819	0.03177	0.26994	0.00100	0.00354	0.00000	0.01106	0.04067	0.02976	0.12723	0.26240	0.08354	0.04746

Table S2. Full results of permutation importance analyses for our ML models.
